# Specificity and recognition of the ADP-ribosyl-ubiquitin modification in the DNA damage response

**DOI:** 10.1371/journal.pbio.3003747

**Published:** 2026-04-02

**Authors:** Chatrin Chatrin, Kang Zhu, Michael D. R. Simmons, Lucy Maginn, Kira Schützenhofer, Yang Lu, Nina Đukić, Sven Wijngaarden, Max S. Kloet, Katarzyna Wiktoria Kliza, Gerbrand J. van der Heden van Noort, Dmitri V. Filippov, Dragana Ahel, Rebecca Smith, Ivan Ahel

**Affiliations:** 1 Sir William Dunn School of Pathology, University of Oxford, Oxford, United Kingdom; 2 Health Science Center, East China Normal University, Shanghai, China; 3 Leiden Institute of Chemistry, Leiden University, Leiden, The Netherlands; 4 Department of Cell and Chemical Biology, Leiden University Medical Centre, Leiden, The Netherlands; 5 Max Planck Institute of Molecular Physiology, Dortmund, Germany; 6 St Vincent’s Institute of Medical Research, Fitzroy, Victoria, Australia; The Univ. of Texas at Austin, UNITED STATES OF AMERICA

## Abstract

ADP-ribosylation (ADPr) is a post-translational modification that has regulatory roles in multiple cellular pathways including the DNA damage response and in innate immunity. Recently, it has been uncovered that ADP-ribose can be further modified by a family of ubiquitin E3 ligases, the DELTEXES, which catalyze ubiquitin transfer directly onto ADP-ribose, creating a hybrid ADPr-Ub modification which can be recognized by proteins with dedicated ADPr-Ub binding domains. With this hybrid modification recently been identified in cellular systems, we use a series of in vitro and cellular assays in human cells to investigate the amino acid preference for ADPr-Ub production as well as conditions required for reversal of the modification. We show that ADPr on both serine and glutamate-linked peptides can be ubiquitinated by the RING-DTC domains of DTX2 and DTX3L in vitro and that this can be recognized by RNF114, RNF138 and RNF166 for ubiquitin chain elongation. Finally, we demonstrate that DTX2 rather than DTX3L plays a role in ADPr-Ub production at sites of DNA damage to promote the recruitment of RNF114, RNF138, and RNF166 in an HPF1-independent manner.

## Abstract 2

Recent studies have shown that ADP-ribose modifications can be further modified by ubiquitin (ADPr-Ub), but there are still major gaps in our knowledge around this modification and its role in the DNA damage response. This study biochemically characterises the amino acid specificity for ADPr-Ub production and its recognition by E3 ubiquitin ligases.

## Introduction

Post-translational modifications (PTMs) are fundamental regulatory mechanisms that dynamically control protein stability, activity, localization, and interactions. Two major PTMs—**ubiquitination** and **ADP-ribosylation** (ADPr)—are involved in numerous cellular processes including DNA repair, immune signaling, and protein quality control [1–3]. ADPr is catalyzed by the ADP-ribosyltransferase superfamily of enzymes including the 17-member PARP family in humans. Of this family, 15 PARPs are catalytically active for ADP-ribosyltransferase activity and transfer ADP-ribose units from NAD+ onto specific amino acid residues—modulating protein function in a highly regulated manner [2,4]. Amino acid specificity of different PARPs can vary depending on the exact PARP family member or on other regulatory proteins [5,6]. For example, Serine-ADPr (Ser-ADPr), the most robust form of ADPr in the DNA damage response, is mediated by PARP1 or PARP2 proteins but only in the presence of the accessory factor HPF1—which is required to deprotonate the hydroxyl of the Serine residues [7–10]. PARP1 and PARP2 can also target glutamate and aspartate residues which can proceed independent of HPF1 and this likely regulates distinct aspects of the DNA damage response as well as gene expression and other processes [11–15]. As with most PTMs, ADPr is a mostly reversible modification which is mediated by different hydrolase enzymes [16].

Ubiquitination regulates many processes including protein turnover, immunity and DNA damage responses [1]. Ubiquitination is catalyzed in a three-step cascade where ubiquitin is first activated in an ATP-dependent manner by an E1-activating enzyme [17–19], received onto an E2-conjugating enzyme and transferred onto a substrate, primarily lysine residues, through the action of one of over 600 E3-ubiquitin ligases. Along with catalyzing mono-ubiquitination, E3 ligases can initiate and extend ubiquitin chains, predominantly nucleating from lysine residues of the acceptor ubiquitin—creating a “ubiquitin code”. The combination of ubiquitin chain linkages and branching events enables the ubiquitin code to specifically regulate a diversity of cellular pathways [1].

Recently, the ubiquitin code has expanded beyond canonical lysine-linked ubiquitination with the identification of the ubiquitination of Ser/Thr residues [20–22], polysaccharides [23], and nucleic acids [24,25]. Notably, ubiquitin can also form a linkage to the 3′-OH the adenosine proximal ribose of ADPr linked either to proteins or nucleic acids creating a dual hybrid PTM—ADP-ribose ubiquitin (ADPr-Ub) [26–30]. ADPr-Ub is also referred to as MARUbe when produced on mono-(ADP-ribose) [31] and is catalyzed by the DELTEX (DTX) family of E3 ubiquitin ligases (DTX1, 2, 3, 3L and 4) [27,28]. Mechanistically, the RING domain in these E3s accommodates E2~Ub while the DTC domain accommodates ADP-ribose/NAD+ [32,33] and contains a catalytic His/Glu dyad that increases the nucleophilicity of the 3′-OH of the adenosine-proximal ribose of ADPr to enable the transfer of ubiquitin from the E2 onto ADPr [28]. Reversal of ADPr-Ub was shown to be performed by several ADPr and ubiquitin hydrolases [27,28]. While ADPr-Ub was initially discovered using biochemical approaches [28], this dual modification has now been identified in cells on tankyrase [34] and PARPs 7 and 10 [31,35] utilizing overexpression systems and has been proposed to regulate protein degradation or in case of tankyrases—protein stabilization. In addition, ADPr-Ub marks have recently been detected on PARP1 and histones in an endogenous system [36]; however, the physiological consequences of this are not clear. It was proposed that these marks may be important for the recruitment of readers such as RNF114 to sites of DNA damage [29]. Besides from the DNA damage response, ADPr-Ub is also potentially involved in CreBP transcription and AR/AHR signaling [30,35,37,38].

Just like ubiquitin, it was recently shown that ADPr-Ub can be extended in some situations to form ubiquitin chains [26]. This was initially shown for the ADPr-Ub reader E3 ubiquitin ligase RNF114 using synthetic substrates [29]. Other studies have shown specific extension of ADP-Ub made by PARP7 and tankyrase via the action of RNF114 and its paralogue RNF166 [34,39]. The study by Kloet *and colleagues* utilized a chemically synthesized ADPr-Ub probe and identified RNF114, RNF138, RNF166, and several other proteins as potential readers of ADPr-Ub [29]. RNF114, RNF166, and a number of other readers were then revealed using complementary methods by Lacoursiere *and colleagues* [39]. Importantly, RNF114 is recruited to sites of DNA damage in a PARP1-dependent manner by its tandem ADPr (ZnF2 + 3) and ubiquitin (UIM) binding domains [29] suggesting that ADPr-Ub may be found at the sites of DNA damage. Mutating either the ZnF2 + 3 or UIM domains leads to reduced RNF114 recruitment [29,40]. Interestingly, treatment with nimbolide which hinders RNF114 substrate recognition [41], promotes PARP1 trapping [42].

The identification of ADPr-Ub is a very recent discovery and while initial biochemical characterization has been made, there are still major gaps in the basic knowledge around this modification. To better understand ADPr-Ub, we completed a series of biochemical assays with synthetic peptides and probes to investigate the amino acid preference for ADPr-Ub production. Furthermore, we examined the reversal of the ADPr-Ub using both ADP-ribosylhydrolases (ARHs) and deubiquitinases (DUBs). Finally, we explored the recruitment of RNF114/138/166 to sites of DNA damage, identifying a dependency on DTX2 for their efficient enrichment at break sites. Importantly, we also show that RNF114 recruitment does not require HPF1 indicating that the enrichment of the ADPr-Ub binding E3 ligases at sites of damage is largely independent of Ser-ADPr.

## Results

### Deltex ubiquitin ligases can ubiquitinate ADP-ribose linked to glutamate and serine residues

The Deltex family of E3 ubiquitin ligases can ubiquitinate ADP-ribosylated substrates in vitro [27,28,35], but to date there is little significant data on physiological ADPr-Ub sites. Recently cysteine-linked ADPr has been shown to be an acceptor of ubiquitin in the context of AR/AHR signaling [30,35], while serine-linked ADPr was shown to be an acceptor of ubiquitin in cells in the context of DNA damage response, specifically on PARP1 (Ser499), histone H2B (Ser6) and H3 (Ser10) [36]. These three sites represent the most robustly ADP-ribosylated sites upon DNA damage [7,8,43]. To provide new insights into the mechanisms, specificity and regulation of ADPr-Ub formation we utilized a synthetic H2B Ser-ADPr peptide to reconstitute ADPr-Ub reactions in vitro. As improvements in Mass Spectrometry sample preparation have elucidated that there is a significant amount of glutamate linked ADPr synthesized during the DNA damage response [13,15,44], we also analyzed the major glutamate-ADPr site on PARP1, E491. Previously, the RING-DTC domains of DTX2 and DTX3L have been shown to be sufficient for the ubiquitination of ADP-ribose [28]. Using these domains in our established in vitro ADPr-Ub assay [28], we tested whether the two DELTEX E3 ubiquitin ligases implicated in the DDR, DTX2, and DTX3L [45,46], show a preference for either of these two sites using two ADP-ribosylated synthetic peptides—Ser-ADPr and Glu-ADPr.

**Fig 1:**
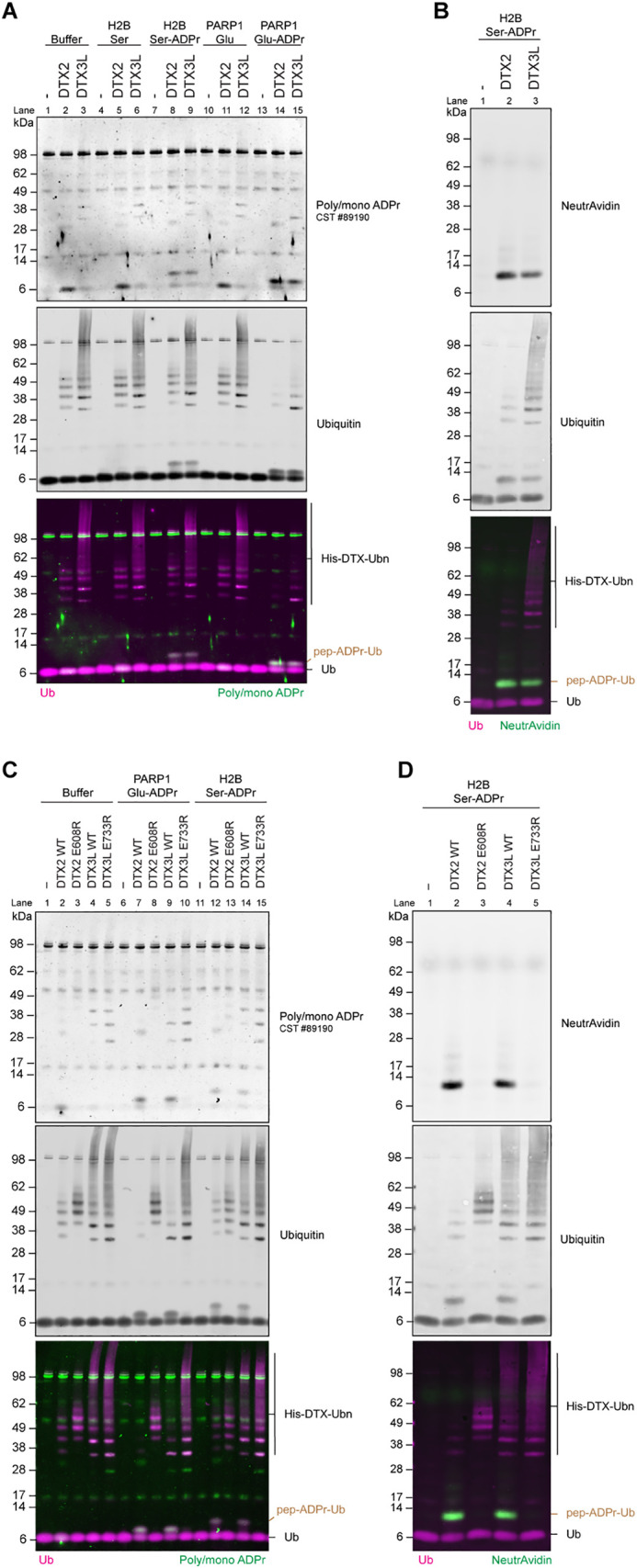
DTX2 and DTX3L modify ADP-ribosylated H2B and PARP1 peptides with ubiquitin. **(A)** PARP1 Glu-ADPr, H2B Ser-ADPr and **(B)** biotin-H2B-Ser-ADPr peptides reacted with wild-type DTX2(RING-DTC) and DTX3L(RING-DTC) and their DTC mutants **(C, D)**. All reactions contain E1, UbcH5b, Ub, MgCl_2_, ATP. ADP-ribosylated peptides were detected with antibodies for ubiquitin or poly/mono-ADPr, or Neutravidin800. Auto-modified DTX is indicated as His-DTX-Ubn on the merged image. ADPr-Ub on peptide substrates are indicated as pep-ADPr-Ub on the merged images.

For both peptides and E3s, we observed a mobility shift in ubiquitin staining that colocalizes with poly/mono-ADPr staining—suggesting a covalent linkage between the ADP-ribosylated peptide and ubiquitin (Fig 1A, Lanes 8–9 and 14–15, S1A Fig). Importantly, the corresponding synthetic peptides that lack ADPr were not modified with ubiquitin (Fig 1A, Lanes 5–6 and 11–12). All samples displayed apparent ubiquitin auto-modification on Deltexes, indicating that these E3 ligases were active in all conditions (Fig 1A, Lanes 2–3, 5–6, 8–9, 11–12, and 14–15). ADPr was detected with an antibody which recognizes poly- and mono-ADPr and the signal coincided with the ubiquitin signal. Notably, we detected some unspecific signal using this antibody at the size of ubiquitin, particularly in the case of DTX2. However, this is also seen with non-ADPr substrates and likely represents a cross-reaction with some product of the ubiquitination reaction components on ubiquitin. To more clearly show ubiquitination of ADPr, we utilized a variant of the H2B Ser-ADPr synthetic peptide with a biotin group and performed the ubiquitination reaction with DTX2 and DTX3L RING-DTC domains (Fig 1B). Using NeutrAvidin to recognize biotin, we detected a single band specifically in the reactions with DTX2-RD and DTX3L-RD (Fig 1B Lanes 2–3), confirming previous data that indicates these E3 ligases ubiquitinate modified peptides [28]. To gain understanding on whether DTX2 or DTX3L prefers Ser-ADPr or Glu-ADPr, we performed a titration with increasing amounts of DTX2-RD or DTX3L-RD (S1B Fig). Each enzyme efficiently ubiquitinated both substrates, with a slight preference to Glu-ADPr under these experimental conditions.

Next, we analyzed the effect of DTX2 and DTX3L DTC domain mutants—E608R and E733R, respectively—that are specifically needed for non-canonical ADP-ribose-linked ubiquitination [28]. Using WT proteins, we observed strong ubiquitination on the peptides H2B-Ser-ADPr, PARP1-Glu-ADPr, and biotin-H2B-Ser-ADPr (Fig 1C Lanes 7, 9, 12, 14; Fig 1D Lanes 2, 4; S1A Fig). This activity was absent for the DTC mutants (Fig 1C Lanes 8, 10, 13, 15 and Fig 1D Lanes 3 and 5). Interestingly, DTX auto-modification is enhanced with these DTC mutants, demonstrating that ADPr-Ub reactions efficiently outcompete DTX auto-ubiquitination in WT proteins.

Taken together, this data suggests that the RING-DTC domain of DTX2 and DTX3L possess the ability to preferentially modify ADPr peptides, but do not discriminate the amino acids through which ADPr is linked.

### ADPr hydrolases efficiently remove ADPr-Ub

**Fig 2:**
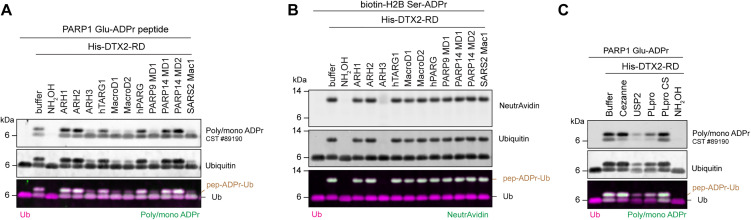
ADPr-Ub is efficiently reversed by ADP-ribosylhydrolases. **(A)** PARP1 Glu-ADPr and **(B)** biotin-H2B Ser-ADPr peptides ubiquitinated by DTX2-RD are further treated with various ADPr-hydrolases and **(C)** deubiquitinases. All reactions contain E1, UbcH5b, Ub, MgCl_2_, ATP. ADP-ribosylated peptides were detected with antibodies for ubiquitin or poly/mono-ADPr, or neutravidin800. ADPr-Ub on peptide substrates are indicated as pep-ADPr-Ub on the merged images.

ADPr and ubiquitination are reversable PTMs. Each system has developed a set of enzymes that are responsible for their efficient reversal. In humans, ADPr reversal is carried out by two families of proteins—the ARHs and hydrolytic macrodomains [16]. While the specificity of these hydrolases for individual amino acid-ADPr linkages is well characterized, it is less clear whether ADPr hydrolase capabilities extend to the removal of ADPr-Ub. Serine-linked ADP-ribose is reversed by ARH3 [47] while glutamate-linked ADP-ribose can be reversed by several hydrolytic macrodomains including MacroD1, MacroD2, TARG1, PARP9 macrodomain 1, and PARP14 macrodomain 1 [15,48–51]. In vitro data has also suggested ARH3 can reverse Glu-ADPr [52]. Previous data has shown that none of these hydrolases can cleave the ADPr-Ub bond [28]. To determine if each hydrolase can remove ADPr-Ub *en bloc* from peptides, we first produced ADPr-Ub on both PARP1-Glu-ADPr and H2B-Ser-ADPr peptides using DTX2-RD. We then treated these samples with different hydrolases to determine which could efficiently reverse ADPr-Ub (S2A Fig). Glu-ADPr-Ub was efficiently reversed by ARH3, MacroD1, MacroD2, and PARP9 macrodomain 1 while the hydrolytic activity on Glu-ADPr-Ub by TARG1 and PARP14 macrodomain 1 was weak (Fig 2A). Glu-ADPr-Ub was not reversed by ARH1, the hydrolase previously shown to be active specifically on Arg-ADPr [53] or by ARH2 or PARP14 macrodomain 2 which have not been shown to possess hydrolytic activity [2]. Glu-linked ADPr-Ub was also reversed by SARS2 Mac1, the hydrolytic macrodomain within SARS-CoV-2 non-structural protein 3 (Nsp3) which has been shown to demonstrate Glu-ADPr hydrolytic activity [49]. Both Ser-ADPr-Ub and Glu-ADPr-Ub were reversed by hydroxylamine treatment (NH_2_OH) which attacks the ester bond of Glu-ADPr but also the ester linkage between ADPr-Ub releasing free ubiquitin instead of ADPr-Ub and thus producing a slightly faster-moving ubiquitin blot signal (Fig 2A and 2B). Ser-ADPr-Ub on the other hand was specifically reversed by ARH3 which is the only described ADPr-hydrolase that is able to hydrolyze the Ser-linked ADPr (Fig 2B) [47]. To ensure the lack of reversal was not due to inactive proteins, we tested the activity of PARP14 MD1, SARS2 Mac1, and TARG1 to reverse Glu-ADPr from a peptide substrate (S2B Fig). Similarly, we show that ARH1 is active through reversal of Arg-ADPr model substrate (S2C Fig). Finally, we show that PARG is active on poly(ADP-ribosyl)ated PARP1 (S2D Fig).

In addition to hydroxylamine treatment, ADPr-Ub can potentially be reversed by certain DUBs. To determine if DUBs could also reverse ADPr-Ub, we treated Glu-ADPr-Ub with Cezanne (a DUB specific for K11 linked ubiquitin) [54], USP2 (a non-specific DUB) [55], and active and inactive PLpro (the SARS-CoV-2 protease found in NSP3) [28]. USP2 could efficiently reverse Glu-ADPr-Ub. PLpro also exhibited some activity on ADPr-Ub while Cezanne and inactive PLpro did not reverse the ubiquitination on Glu-ADPr (Figs 2C and S2E).

Together, this data suggests that ADPr-Ub can be efficiently removed *en bloc* by several ADP-ribosyl hydrolases provided that the peptide-ADPr linkage fits their substrate specificity. Alternatively, as certain DUBs displayed activity on ADPr-Ub, hydrolysis could begin with DUBs and be followed by a specific ADPr hydrolase to achieve complete ADPr-Ub removal.

### RING-UIM E3 ligases can extend ubiquitin chains on ADPr-Ub

**Fig 3:**
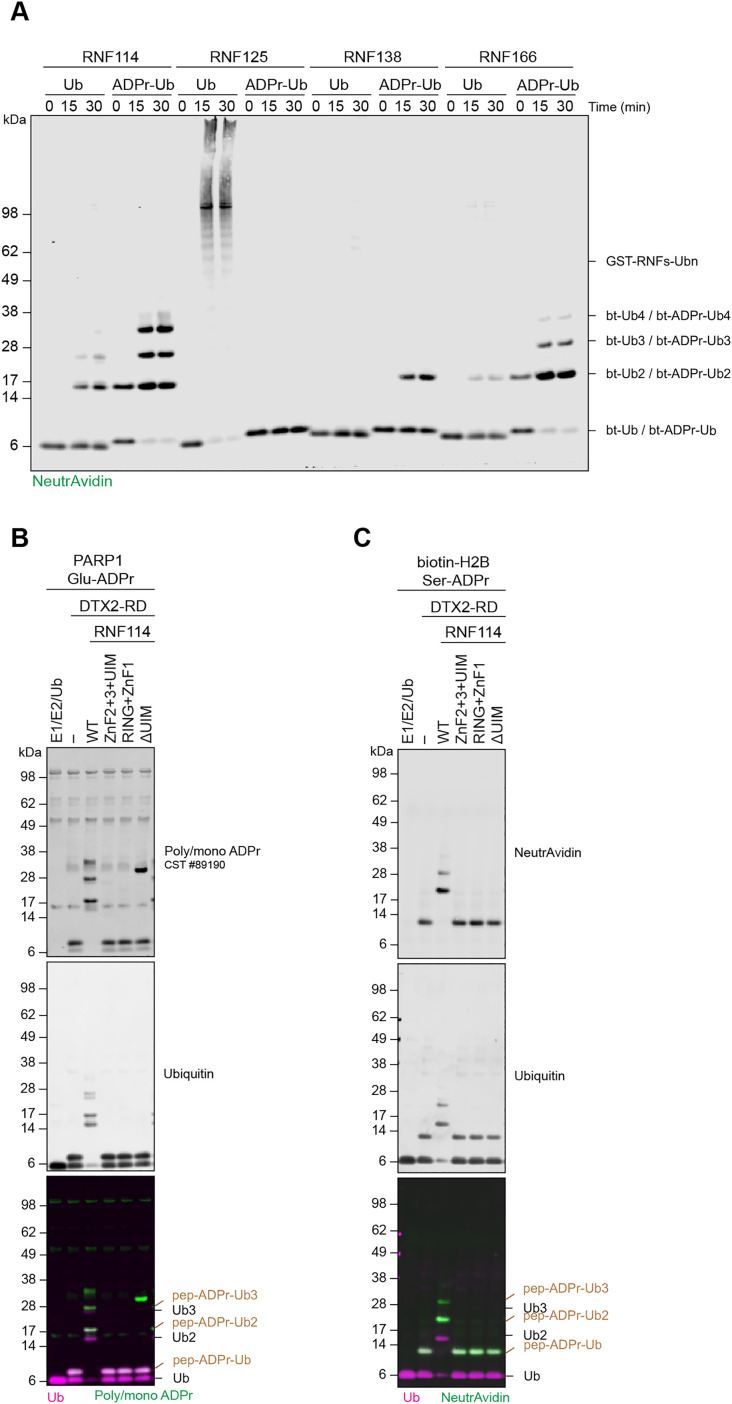
RING-UIM E3 ligases can extend ubiquitin chains on ADPr-Ub. **(A)** Biotin-ADPr-Ub or biotin-Ub elongation by RING-UIM E3s. **(B)** ADPr-Ub elongation on Glu-ADPr and **(C)** biotin-Ser-ADPr peptides by RNF114 variants. All reactions contain E1, UbcH5b, Ub, MgCl_2_, ATP. ADP-ribosylated peptides were detected with antibodies for ubiquitin or poly/mono-ADPr, or Neutravidin800. ADPr-Ub on peptide substrates are indicated as pep-ADPr-Ub on the merged images.

Recently, it was shown that RNF114 can act as a reader of ADPr-Ub and further extend ADPr-Ub with K11 linked poly-ubiquitin chains [29]. RNF114, together with RNF125, RNF138 and RNF166, is a member of the RING-UIM family of ubiquitin ligases that is characterized by an N-terminal C3HC4 RING domain, three Zinc finger (ZnF) domains and a C-terminal ubiquitin-interacting motif (UIM). Modeling and functional data suggested that ZnF2-3 together with the UIM are binders of ADPr-Ub [29]. More recently, this was confirmed for RNF114 and another member of the family RNF166 [34,39]. Given that RNF138 and RNF166 were significantly enriched by a biotin-ADPr-Ub probe [29], we compared all four RING-UIM E3 ligases for their ability to extend ubiquitin chains on the model ADPr-Ub substrate. Using a biotin-Ub or biotin-ADPr-Ub probe that was previously used to identify RNF114 as a ADPr-Ub binder [29], we saw that RNF114 along with RNF138 and RNF166 could efficiently elongate the ubiquitin chains with a preference for the ADPr-Ub probe over auto-ubiquitination (Figs 3A and S3A). RNF125 displayed auto-ubiquitination but failed to display any ubiquitin chain extension on the ADPr-Ub probe. We also observed that RNF114 and RNF166 form protein free poly-ubiquitin chains, similar to what was previously seen [29,39]. Finally, to check whether ubiquitin chain extension by RNF114 has an ADPr-Ub amino acid preference, we performed ADPr-Ub chain extension on Glu-ADPr-Ub and Ser-ADPr-Ub peptides. As seen with the biotin-ADPr-Ub probe, RNF114 could efficiently extend ubiquitin chains on both peptides (Figs 3B, 3C, and S3B). Furthermore, we confirmed that full-length RNF114 is required for extension. The RNF114 ZnF2-3 + UIM, the ADPr-Ub binding domain, could not extend ubiquitin chains on ADPr-Ub, nor could RNF114 RING-ZnF1 or RNF114 with a deletion of the UIM domain. This data, consistent with our previous work, supports that the binding of RNF114 to ADPr-Ub is a requirement for ubiquitin chain elongation.

### DTX2 drives recruitment of RING-UIM E3 ligases to sites of damage

**Fig 4:**
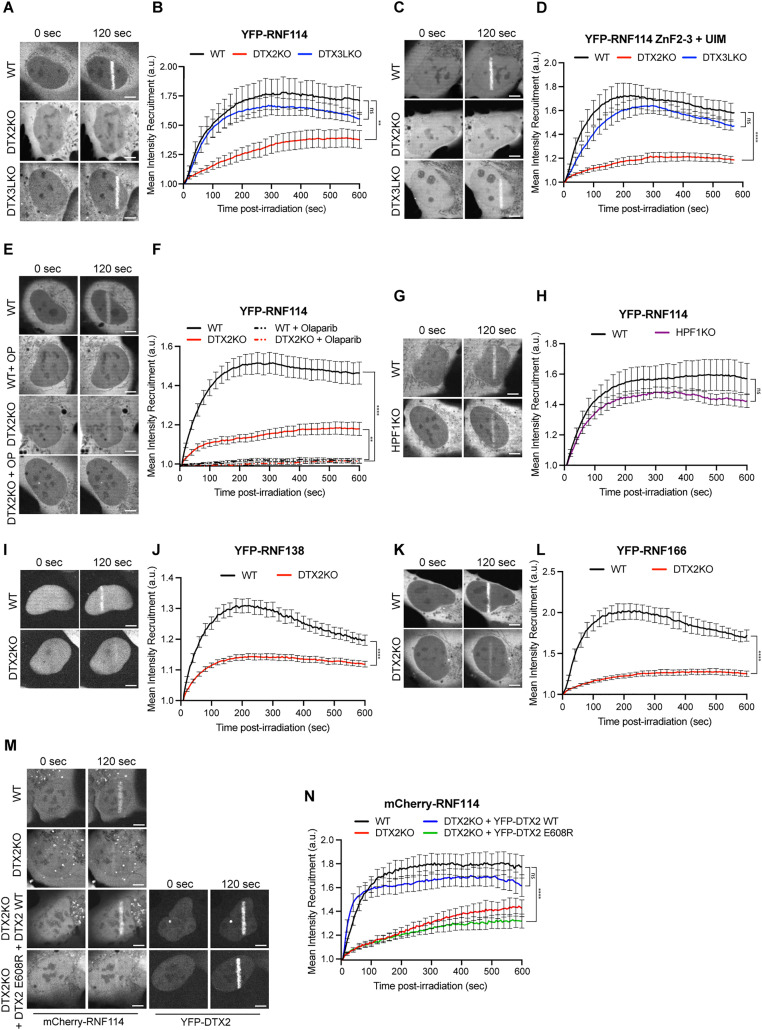
DTX2-dependent ADPr-Ub drives recruitment of RING-UIM E3 ligases to sites of damage. **(A)** Representative confocal images and **(B)** recruitment kinetics of full-length YFP-RNF114 recruitment to sites of DNA damage induced by 405 nm irradiation in U2OS WT (Black), DTX2KO (Red), or DTX3LKO (Blue) cells. **(C)** Representative confocal images and **(D)** recruitment kinetics of YFP-RNF114 ZnF2-3 + UIM recruitment to sites of DNA damage induced by 405 nm irradiation in U2OS WT (Black), DTX2KO (Red), or DTX3LKO (Blue) cells. **(E)** Representative confocal images and **(F)** recruitment kinetics of full-length YFP-RNF114 recruitment to sites of DNA damage induced by 405 nm irradiation in U2OS WT (Black) or DTX2KO (Red) treated or not with 1 μM Olaparib. **(G)** Representative confocal images and **(H)** recruitment kinetics of full-length YFP-RNF114 recruitment to sites of DNA damage induced by 405 nm irradiation in U2OS WT (Black) or HPF1KO (Purple) cells. **(I)** Representative confocal images and **(J)** recruitment kinetics of full length YFP-RNF138 recruitment to sites of DNA damage induced by 405 nm irradiation in U2OS WT (Black) or DTX2KO (Red) cells. **(K)** Representative confocal images and **(L)** recruitment kinetics of full-length YFP-RNF166 recruitment to sites of DNA damage induced by 405 nm irradiation in U2OS WT (Black) or DTX2KO (Red) cells. **(M)** Representative confocal images and **(N)** recruitment kinetics of full-length mCherry-RNF114 recruitment to sites of DNA damage induced by 405 nm irradiation in U2OS WT (Black), DTX2KO (Red) cells, and DTX2KO cells complemented with YFP-DTX2 WT (Blue) or E608R (Green). For all images, Scale bar, 5 µm. Data is representative of at least 3 independent replicates. Curves show mean ± s.e.m. of between 8 and 20 cells per condition. *P* values were obtained using an unpaired Student *t* test with Bonferroni correction (ns, not significant, **P < 0.01,****P < 0.001). The data for this figure can be found at https://www.ebi.ac.uk/biostudies/studies/ S-BIAD2514.

ADPr is one of the earliest detectable PTMs at sites of DNA damage. Previously, RNF114 was suggested to be recruited to DNA breaks through an interaction with mono- or poly-ADPr [40,42]. However, our recent work challenged this, suggesting that it was instead an interaction with ADPr-Ub that drives the recruitment of RNF114 to break sites [29]. Loss of either the ZnF2-3, which bind ADPr, or the UIM was sufficient to inhibit recruitment of RNF114 to break sites. Given this, we wanted to determine if loss of DTX2 or DTX3L which catalyze ADPr-Ub would modulate the recruitment of RNF114 to break sites. Comparing the recruitment of YFP-RNF114 to sites of damage in DTX2 or DTX3L knockout U2OS cells (S4A Fig) to their WT counterpart, we saw that there was a marked reduction in YFP-RNF114 accumulation at breaks in DTX2KO cells (Figs 4A, 4B, and S4B). Moreover, we saw that loss of DTX2 reduced the recruitment of the minimal ADPr-Ub binding domain of RNF114 (Znf2-3+UIM) in a similar way, with recruitment in DTX3LKO cells reaching similar levels as that of WT cells (Figs 4C, 4D, and S4C). Next, we treated both WT and DTX2KO cells with olaparib to inhibit ADPr production at sites of damage. Inhibition of PARP1 and PARP2, the major producers of ADPr at break sites, abolished RNF114 recruitment in both WT and in DTX2KO cells (Figs 4E, 4F, and S4D). Importantly, we compared ADPr signals after DNA damage induction in DTX2KO and DTX3LKO cells by western blotting. Here we did not observe any notable changes in the pattern of ADPr with three different ADPr antibodies suggesting reduced RNF114 recruitment in DTX2KO cells is not due to significant changes in ADPr signaling (S4E Fig). Together, this confirms that the ADPr activity of PARP1/2 is essential for the recruitment of RNF114, and that there is an added level of specificity and binding affinity where DTX2 rather than DTX3L is required for the production of ADPr-Ub at sites of damage.

As our in vitro data showed that recombinant DTX2 RING-DTC domain do not show a preference for either Ser- or Glu-linked ADPr, we wanted to assess whether there is any specificity in cells. For this, we compared the recruitment of RNF114 in WT or HPF1KO cells where the major form of DNA damage-associated ADPr is Ser- or Glu-linked, respectively (Figs 4G, 4H, and S4F). HPF1 is essential for synthesis of serine-linked ADPr in cells [7,43]. Here, we saw that there was no significant difference in YFP-RNF114 recruitment in WT or HPF1KO cells, suggesting that in a cellular context, serine-linked ADPr-Ub is not a major or dominant driver of recruitment.

As RNF138 and RNF166 demonstrated a similar ability to extend Ub chains on ADPr-Ub as RNF114 and contain a ZnF2-3 + UIM domain for ADPr-Ub binding, we compared their recruitment to sites of damage in WT or DTX2KO cells. Here, as with RNF114, we saw a dependency on DTX2 to drive efficient recruitment of RNF138 (Figs 4I, 4J, and S4G) and RNF166 (Figs 4K, 4L, and S4H) to sites of DNA damage.

Finally, to confirm the recruitment of RNF114 to sites of DNA damage is reliant on the production of ADPr-Ub by DTX2, we complemented DTX2KO cells with YFP-DTX2 WT or the DTC mutant E608R. While DTX2 WT efficiently restored RNF114 recruitment to levels observed in WT cells, the DTC mutant failed to restore RNF114 recruitment (Figs 4M, 4N, and S4I Importantly, DTX2 E608R still efficiently recruited to sites of damage, suggesting that reduced RNF114 recruitment to breaks is due to deficiency in the catalytic activity of this mutant and not due to reduced DTX2 recruitment to sites of damage.

Together, this data suggests that ADPr-Ub at the sites of DNA damage is initiated by PARP1/2 mediated ADPr which is subsequently extended in a DTX2 dependent manner to form ADPr-Ub and this dual PTM acts to recruit three different RING-UIM E3 ligases to sites of DNA damage.

## Discussion

The recent discovery of ADPr-Ub [3,26,28] has opened a new area of research at the intersection between ADPr and ubiquitin signaling. Recent efforts from a number of groups have aimed at trying to develop tools that will allow for the clarification of ADPr-Ub specificity, the identification of readers and erasers and the phenotypic consequences of ADPr-Ub in cellular systems [29–31,34–36,39].

What has emerged is the beginning of a complicated signaling system that has the potential to be regulated at different levels. Initiation occurs with ADPr of substrates, which is then ubiquitinated by the Deltex family of ubiquitin ligases [28] and this dual PTM can then be further extended by certain members of the RING-UIM E3 ligase family to produce K11-linked ubiquitin chains [29,39], with the potential for different linkages beyond K11 [3]. Given this complexity, it is important to clarify the mechanistic details of this signaling pathway which will facilitate deeper physiological exploration.

The first cellular sites of ADPr-Ub were recently identified in the context of the DNA damage response with the modification found on PARP1 and core histone proteins [36]. Here, ADPr-Ub was found on serine residues, aligning with what is known about ADPr in DNA repair where serine residues are the major ADPr acceptors [8] and the previous demonstration that serine sites on histones are modified by DTX2 in vitro [28]. Knowing this, we used one of these DNA damage-inducible sites as a model substrate to reconstitute biochemical reactions using recombinant proteins to assess specificity. In addition to Histone H2B S6, we also assessed a Glu-ADPr site of PARP1, E491, which is a major acceptor of ADPr after DNA damage induction and was recently identified in cells as a result of improvements in the preservation of ester-linked ADPr making it more amenable for mass spectrometry [12,13,15,44]. Our work using the minimal catalytic fragments of Deltex recombinant enzymes required to perform ADPr-Ub, the RING-DTC, from DTX2 and DTX3L which represent the two different subgroups within the Deltex family [24], show that they may not display efficient discrimination between Ser- and Glu-linked ADPr sites in vitro (Figs 1 and S1).

Deltex ubiquitin ligases can perform both ADPr-Ub and direct auto-ubiquitination. Comparing WT and DTC ADPr-binding deficient mutants of DTX2 and DTX3L [32], we observed an increase in auto-ubiquitination and a complete absence of ADPr-Ub production when the DTC was mutated (Fig 1C and 1D) supporting the notion that the DTC domain preferentially directs the activity of the RING domain towards modifying ADPr substrates.

To date, the reversal of ADPr-Ub has not been thoroughly investigated. In this current report, we confirm this activity on the physiologically relevant H2B Ser6 site, showing that ADPr-Ub can be removed *en bloc* by ARH3 This result is compatible with the structure of ARH3 that suggests the accessible active site of ARH3 can accept adducts on the ADPr proximal ribose group [28,56]. Given that ARH3 is the only reported ADP ribosylhydrolase with activity against serine-ADPr [47], it is not surprising that ADPr-Ub can only be removed *en bloc* by ARH3 (Fig 2B). Our data also shows that the majority of Glu-targeting ARHs can at least to some extent hydrolyze Glu-ADPr-Ub on a peptide derived from the PARP1 automodification domain (Fig 2A). Thus, while the ubiquitin component of the modification can be removed by several human, viral and bacterial DUBs or proteases (Fig 2C, [28,39]) and the subsequent mono-ADPr reversed by ADPr hydrolases, our data suggests that this intermediate protease step may not always be required for certain macrodomains or ARH3 to reverse ADPr-Ub *en bloc*.

Initial reports have suggested that ubiquitination of ADPr by the Deltexes can be a priming event for further elongation of ubiquitin chains with RNF114 being shown to extend K11 linked ubiquitin chains on ADPr-Ub [29]. In this report, we show that two additional RNF114-like E3 ligases, RNF138, and RNF166, can extend ubiquitin chains on ADPr-Ub (Fig 3A). As supported by a recent report from Cohen and Pruneda [39], RNF114, RNF138, and RNF166 show preferential extension of Ub chains on ADPr-Ub compared to Ub (Fig 3B and 3C). Notably, ADP-Ub extension activity was not displayed by the final family member RNF125, despite it showing auto-ubiquitination activity and possessing the same domain architecture.

In our in vitro work we used a simplified representation of DTX proteins utilizing the minimal fragment required for ADPr-Ub (RING-DTC); however, we recognize the possibility that full-length DTX protein containing the additional domains (e.g., KH or WWE) [27,28] may increase the specificity and efficiency of this modification. To assess this as well as the potential wider contributions to specificity from the cellular environment and protein complexes, we investigated the role of Deltexes and ADPr-Ub at sites of DNA damage in cells. Initial reports have suggested that ADPr-Ub can be found on DNA damage-induced Ser-ADPr sites, specifically histones and PARP1 [36]. While RNF114, RNF138 and RNF166 have been reported to recruit to damage [40,57,58], with RNF114 recruitment known to rely on ADPr-Ub binding [29], our data here revealed that the recruitment of these E3 ligases is reliant on DTX2-dependent ADPr-Ub as loss of DTX2 greatly reduced the recruitment of full-length, or the ADPr-Ub binding domains, of these E3 ligases to sites of damage (Fig 4). Furthermore, complementation of a DTC mutant of DTX2 that cannot complete the ADPr-Ub reaction fails to rescue RNF114 recruitment to sites of damage. The recruitment of RNF138 is aligned with its proposed roles in the DDR and with previous data showing that the loss of ZNF2/3 reduce its recruitment to sites of damage, similar to what was observed for RNF114 [29,57]. However, the same was not observed upon loss of DTX3L, suggesting that DTX2 may be the major regulator of ADPr-Ub in a DNA damage context. This is fitting to what is known about DTX2, with it being a nuclear protein with previous links to DNA repair [33,45].

The recruitment of RNF114 to sites of DNA damage was suggested to rely on HPF1-dependent mono-Ser-ADPr, where overexpression of HPF1 resulted in an increase in RNF114 recruitment to DNA breaks [40]. Importantly, we did not observe changes to RNF114 recruitment in WT or HPF1KO cells where the major form of DNA damage induced ADPr is Ser- and Glu-, respectively (Fig 4G and 4H) [8]. This suggests that serine-linked ADPr may not be the dominant modification site for DTX2-dependent modification, which is required for RNF114 recruitment. Thus, our data suggests a surprising twist highlighting the importance of Glu/Asp-ADPr or even DNA-ADPr or Tyrosine linked-ADPr at sites of damage as a means of redundancy [59–62].

Altogether, our data clarifies several important aspects of ADPr-Ub signaling in DDR; DTX2 catalyzes the production of ADPr-Ub at sites of damage that can act as a binding platform for specific readers of the hybrid modification and that the production of ADPr-Ub and that the production of ADPr-Ub is not reliant on HPF1 dependent Ser-ADPr. The data here also provides a platform and directions for more efficient research in the future on this emerging and complex signaling pathway [3,26].

## Methods

### Cloning

Constructs were generated using standard polymerase chain reaction (Q5 High-Fidelity DNA polymerase (NEB)) and verified by Sanger sequencing. RNF125, RNF138, RNF166 sequences codon-optimized for *Escherichia coli* were obtained from GenScript. Full-length RNF125, RNF166, and RNF138 (10-245) were cloned into a modified pGEX4T-3 vector with an N-terminal GST tag followed by a TEV cleavage site [32]. Full length or truncated RNF114, RNF138, and RNF166 were cloned into pDEST-YFP [63] or pDEST-CMV-N-mCherry [64]. Full-length DTX2 was cloned into pDEST-YFP and the E608R mutation was made using site-directed mutagenesis (Agilent). Constructs for UBA1 (Addgene #34965), UbcH5B, RNF114 [29], DTX2(390-C) [28], DTX3L(544-C) [28], ARH1, ARH2, ARH3, hTARG1, MacroD1, MacroD2, PARG, PARP9 MD1 [49], PARP14 MD1 [49], PARP14 MD2 [49], SARS-CoV-2 (SARS2) Mac1, Cezanne (Addgene #61581, [65]), USP2, PLPro [28] were described previously.

### Protein expression and purification

GST-tagged RNF114/125/138/166 were expressed in *E. coli* Rosetta 2 (DE3) pLysS (Novagen). Cultures were grown in LB medium with appropriate antibiotic at 37 °C. After reaching OD_600_ of 0.6–0.8, 0.2 mM isopropyl-β-D-1-thiogalactopyranoside and 0.1 mM ZnCl_2_ were added, and cultures were grown at 18 °C for 16–18 h. Cells were harvested and resuspended in buffer A (50 mM Tris-HCl pH 8.0, 500 mM NaCl, 1 mM DTT) and supplemented with 0.2 mM phenylmethylsulfonyl fluoride. The resuspended cells were lysed by sonication and cleared by high-speed centrifugation at 39,800  *g* for 1 h at 4 °C. The cleared lysate was incubated with Glutathione sepharose resin (Cytiva), washed in buffer A, and eluted in buffer B (50 mM Tris-HCl pH 8.5, 500 mM NaCl, 5 mM DTT, 10 mM glutathione). Eluate was subjected to size exclusion chromatography on a Superdex 200 column (GE Healthcare) pre-equilibrated in 25 mM HEPES Na pH 7.5, 150 mM NaCl, and 1 mM DTT. Relevant fractions were pooled and concentrated using Amicon ultra centrifugal devices (Merck). Protein concentrations were measured by absorbance at 280 nm on Nanodrop (DeNovix) and calculated based on molar extinction coefficient. Proteins were kept in small aliquots, frozen in liquid N_2_, and stored in −70 °C.

Protocols for generating UBA1, UbcH5B, His-RNF114 and variants, DTX2(390-C), DTX3L(544-C), ARH1, ARH2, ARH3, hTARG1, MacroD1, MacroD2, PARG, PARP9 MD1, PARP14 MD1, PARP14 MD1 G832E, PARP14 MD2, SARS-CoV-2 Mac1, Cezanne, USP2, PLPro, and PARP1 were described previously [9,15,28,29,47,49,56,66,67].

### ADPr-Ub reaction

Fifty µM ADP-ribosylated peptides (Table 1) were incubated with 0.5 µM human UBA1, 2.5 µM UbcH5B, 10 µM ubiquitin, and 2 µM Deltex enzymes (wild-type or mutant His-DTX2-RING-DTC (390-C) or His-DTX3L-RING-DTC (544-C), as indicated) in 50 mM Tris HCl pH 8.0, 50 mM NaCl, 2.5 mM MgCl_2_, 2.5 mM ATP, at 37 °C for 30 min. For S1B Fig, 50 mM Tris HCl pH 8.0 was replaced with 50 mM HEPES Na pH 7.5. For hydrolase treatment (Fig 2), 1 µM of the indicated hydrolases were added, except for Cezanne (100 nM), PLPro wild-type and mutant (5 µM), and hydroxylamine (1 M). Hydrolysis was carried out at 37 °C for 30 min. Reactions were quenched with 3× LDS + 150 mM DTT and incubated at room temperature for 10 min. Synthetic ADPr-Ub for extension by RNF114 and its paralogues was made as previously described [29].

**Table 1:** Peptide used in this study.

Name	Sequence	Source
H2B-S6	H_2_N-PAKSAPAPKKG-CO_2_H	Thermo Fisher
H2B-S6-ADPr	Ac-PAKS[ADPr]APAPKKG-CONH_2_	This Study
Biotin-H2B-S6-ADPr	biotin-PEPAKS[ADPr]APAPKKGSK-CO_2_H	This Study
PARP1-E491	H_2_N-AAPVEVVAPR-CO_2_H	Thermo Fisher
PARP1-E491-ADPr	H_2_N-AAPVE[ADPr]VVAPR-CONH_2_	This Study

Samples were resolved with SDS-PAGE (4%–12% Bis–Tris gel, MES running buffer (Invitrogen)) at 150 V, transferred onto nitrocellulose membrane (Biorad TransBlot Turbo), and blocked with 5% BSA in 0.1% PBS-Tween 20 (PBST). The membrane was incubated with primary antibodies: mouse monoclonal anti-Ub (P4D1) (Santa Cruz Biotechnology sc- 8017), poly/mono-ADP ribose (D9P7Z) rabbit mAb (Cell Signaling Technology #89190), overnight at 4 °C followed by secondary Li-Cor goat-anti mouse IRDye 680, goat-anti rabbit IRDye 800 (Li-Cor Bioscience), NeutrAvidin 800 (Thermo Fisher). Membranes were washed in PBST and scanned using Li-Cor Odyssey CLx imager.

### ADPr-peptide synthesis

The general procedures for solid-phase synthesis of Biotin-PEG3-H2B-S6-ADPr were followed as described by Voorneveld *and colleagues* [68] starting from Tentagel S AC resin (50 μmol scale). Following peptide elongation, Fmoc-PEG_3_-OH (5 eq.) was dissolved in DMF (0.5 M) after which *O*-(1*H*-6-chlorobenzotriazole-1-yl)-1,1,3,3-tetramethyluronium hexafluorophosphate (HCTU, 5 eq.) and diisopropylethylamine (DIPEA, 10 eq.) were added, and the mixture was shaken for 3 min. This activated carboxylic acid was added to the resin (1 eq.), and the mixture was shaken for 1 h. The resin was washed with DMF, after which the Fmoc was deprotected by the addition of 20% v/v piperidine in DMF at 25 °C for 2 × 10 min, followed by extensive washing with DMF. Biotin (5 eq.) was dissolved in DMSO (0.5M) after which HCTU (5 eq.) and DIPEA (10 eq.) were added, and the mixture was shaken for 3 min. The activated biotin was added to the resin after which the mixture was shaken for 1 h, followed by extensive washing with DMF, DCM, and Et_2_O. The ADP-ribose was installed on-resin and the product was cleaved off the resin and purified as described [68]. PARP1-E491-ADPr peptide was prepared as described by Tashiro *and colleagues* [52].

### ADPr-Ub elongation reaction

For Fig 3B and 3C: 50 µM ADP-ribosylated peptides were incubated with 0.5 µM human UBA1, 2.5 µM UbcH5B, 10 µM ubiquitin, 2 µM His-DTX2-RING-DTC (390-C), and 1 µM of His-RNF114 wild-type or mutants in 50 mM Tris HCl pH 8.0, 50 mM NaCl, 2.5 mM MgCl_2_, 2.5 mM ATP, at 37 °C for 30 min. Reactions were quenched with 3× LDS + 150 mM DTT and incubated at room temperature for 10 min. Samples were resolved with SDS-PAGE and western blot as described above.

### Biotin-ADPr-Ub elongation reaction

For Fig 3A: 5 µM of biotin-ADPr-Ub probe [29] or biotin-Ub were incubated with 0.5 µM human UBA1, 2.5 µM UbcH5B, 10 µM ubiquitin, 2 µM His-DTX2-RING-DTC (390-C), and 0.5 µM of GST-RNF114, GST-RNF125, GST-RNF138 (10-245), or GST-RNF166 in 50 mM Tris HCl pH 8.0, 50 mM NaCl, 2.5 mM MgCl_2_, 2.5 mM ATP, at 37 °C for 30 min. Reactions were quenched with 3× LDS + 150 mM DTT and incubated at room temperature for 10 min. Samples were resolved with SDS-PAGE and western blot as described above.

### Cell culture and live-cell imaging

Cell culture and live-cell imaging was completed as previously described [29]. Briefly, human U2OS osteosarcoma (American Type Culture Collection (ATCC HTB-96) WT, DTX2KO, DTX3LKO, [69] or HPF1KO [70] cells were cultured in Dulbecco’s modified Eagle’s medium (Sigma-Aldrich) supplemented with 10% fetal bovine serum (FBS; Gibco) and penicillin-streptomycin (100 U/ml; Gibco) in a humidified atmosphere at 37°C with 5% CO_2_. Cells were plated on an eight-well μ-slide glass bottom chamber slide (ibidi) and transfected with an expression plasmid for YFP-RNF114 WT, YFP-RNF114 Znf2-3 + UIM (amino acid 140−228 of RNF114), RNF138, RNF166 or YFP-RNF166 ZnF2-3 + UIM (amino acids 149−237 of RNF166), mCherry-RNF114 or YFP-DTX2 using TransIT-LT1 Transfection Reagent (Mirus Bio), according to the manufacturer’s instructions. Cells were incubated for 48 hours prior to imaging. For cell sensitization before laser irradiation at 405 nm, growth medium was aspirated from the chamber slide and replaced with fresh medium containing Hoechst 33342 (0.3 μg/ml). Immediately before imaging, the Hoechst-containing medium was replaced with imaging media (phenol red–free Leibovitz’s L-15 medium (Life Technologies) supplemented with 20% FBS, penicillin (100 μg/ml), and streptomycin (100 U/ml)). Live-cell microscopy was carried out on an Olympus IX-83 inverted microscope equipped with a Yokogawa SoRa superresolution spinning-disk head, a UPlanAop 60×/1.5 numerical aperture oil-immersion objective lens and a Prime BSI scientific complementary metal-oxide semiconductor camera. The fluorescence of YFP and mCherry was excited with 488-nm and 561-nm solid-state lasers, respectively, and fluorescence detection was achieved with band-pass filters adapted to the fluorophore emission spectra. Laser microirradiation at 405 nm was performed along a 15-μm line through the nucleus for 250 ms using a single-point scanning head (Olympus cellFRAP) coupled to the epifluorescence backboard of the microscope. To ensure reproducibility, laser power at 405 nm was measured at the beginning of each experiment and set to 110 μW at the sample level. For time course experiments, images were collected every 5 s. For the live-cell imaging experiments, cells were maintained at 37 °C with a heating chamber. Protein accumulation at sites of damage (*Ad*) was then calculated as:


$$A_{damage}=\frac{I_{damage}-I_{background}}{I_{nucleus}-I_{background}}$$

The accumulated protein at the damage (Ad) is a measure of the intensity (I) at the sites of damage over the intensity of the entire nucleus, with subtracted background intensity. The intensity within the microirradiated area was then normalized to the intensity before damage induction. Photoactivated H2B was used as a reference to indicate where irradiation had occurred.

For PARPi treatment, cells were incubated with 1 μM Olaparib (Stratech Scientific) for 1 h prior to imaging.

DTX2KO cells were made as previously described [30]

### Cell lysis and western blotting

Cell lysis and western blotting was completed as previously described [49,71]. Briefly, cells were scrapped in PBS and pelleted at 300 *g* for 5 min at 4 °C. PBS was removed and cells were lysed with Triton-X buffer (1% Triton X-100, 100 mM NaCl and 50 mM Tris-HCl, pH 8.0, 5 mM MgCl_2_, 0.1% Benzonase (Sigma Aldrich), 1× protease inhibitor (Roche) on ice for 30 min. Protein samples were quantified using Bradford (BioRad) and equal amounts of protein were boiled in 1× NuPAGE LDS sample buffer (Invitrogen) with 60 mM DTT (Sigma-Aldrich) and resolved on NuPAGE Novex 4%–12% bis-tris gels (Invitrogen) in 1× NuPAGE MOPS SDS Running Buffer (Invitrogen) at 150 V. Proteins were transferred onto nitrocellulose membranes (Bio-Rad) using Trans-Blot Turbo Transfer System (Bio-Rad). The membranes were stained with Ponceau S Staining Solution (Thermo Fisher Scientific) to check the transfer quality, rinsed with water, and blocked in 5% (w/v) nonfat dried milk in PBS buffer with 0.1% (v/v) Tween 20 (PBST) for 1 hour at room temperature. This was followed by overnight incubation with primary antibody as indicated (Table 2) at 4 °C. The next day, membranes were washed in PBST and incubated with HRP-conjugated antibodies at room temperature for 1 hour. Membranes were visualized on Hyperfilm ECL films (Cytiva) after adding Pierce ECL Western Blotting Substrate (Thermo Fisher Scientific).

**Table 2:** Antibodies used in this study.

Target	Host	Company	Reference	Dilution in WB
PARP1	Rabbit	Abcam	Ab32128	1:5,000
DTX2	Rabbit	Novus	NBP2-13941	1:1,000
DTX3L	Rabbit	Santa Cruz	Sc-100627	1:150
HPF1	Rabbit	Novus	NBP1-93973	1:1,000
H3	Rabbit	Millipore	07-690	1:10,000
PAR-ADPr	Rabbit	Merck	MABE1031	1:1,000
β-tubulin	Rabbit	Abcam	Ab6046	1:5,000
Poly/mono-ADPr (D9P7Z)	Rabbit	Cell Signaling	CST-89190	1:1,000
HRP conjugated anti-Mono-ADPr	–	Biorad	AbD43647	1:10,000
HRP-conjugated anti-mouse	Goat	Agilent	P0447	1:3,000
HRP-conjugated anti-rabbit	Swine	Agilent	P0399	1:3,000
Ub (P4D1)	Mouse	Santa Cruz	sc-8017	1:1,000
Goat anti-mouse IgG secondary (680RD)	Goat	Li-Cor Bioscience	926-68070	1:20,000
Goat anti-rabbit IgG secondary (800CW)	Goat	Li-Cor Bioscience	926-32211	1:20,000
NeutrAvidin DyLight 800	–	Thermo Fisher	22853	1:20,000

For DNA damage induction, cell media was replaced with media containing 2 mM H_2_O_2_ for 10 min prior to collection.

### ADP-ribosylhydrolase activity assays

The ADPr-peptides, including arginine- and glutamate-linked ADP-ribosylated substrates used in S2B and S2C Fig for hydrolytic activity analysis, were previously described [72]. The previously established adenosine monophosphate (AMP)-Glo assay was used to analyze the hydrolytic activities of selected mono-ARHs proteins via luminescent detection of ADP-ribose [52]. Briefly, hydrolytic assays were conducted using 10 µM ADP-ribosylated substrates and 500 nM hydrolase in reaction buffer containing 50 mM Tris-HCl [pH 7.5], 200 mM NaCl, 10 mM MgCl_2_, 1 mM DTT, and 0.2 µM Nudix hydrolase 5 (NUDT5) for 30 min at 30 °C. Luminescence resulting from hydrolytic reactions was determined using the AMP-Glo assay kit (Promega) according to the manufacturer’s protocol. Measurements were performed on a SpectraMax M5 plate reader equipped with SoftMax Pro software (Molecular Devices). To account for background, all readings were corrected and normalized against reactions containing NUDT5 but lacking hydrolase, providing a baseline for AMP generated from substrate turnover. Data analysis was conducted in GraphPad Prism, and values were presented as the mean ± standard deviation (s.d.) from three independent replicates.

Poly-ADPr degradation assay were performed as previously described [73]. Briefly, PARP1 automodified using ^32^P-NAD+ as a co-substrate was incubated with recombinant PARG. Reaction products were resolved on SDS-PAGE and visualized by autoradiography.

## Supporting information

S1 Fig DTX2 and DTX3L modify ADP-ribosylated H2B and PARP1 peptides with ubiquitin. **(A)** Uncropped SDS-PAGE of purified recombinant His-DTX2 and His-DTX3L WT and mutant RING-DTC domains. **(B)** PARP1 Glu-ADPr, H2B Ser-ADPr peptides reacted with titrated amounts (0.3, 0.8, 2 µM) of DTX2(RING-DTC) and DTX3L(RING-DTC). Reactions in lanes 3–14 contain E1, UbcH5b, Ub, MgCl_2_, ATP. ADP-ribosylated peptides were detected with antibodies for ubiquitin or poly/mono-ADPr. ADPr-Ub on peptide substrates are indicated as pep-ADPr-Ub on the merged images.
(PDF)

S2 Fig Analysis of human ADP-ribosylhydrolases and DUBs. **(A)** Uncropped SDS-PAGE of purified recombinant ADP-ribosylhydrolases used in this study. **(B, C)** Hydrolytic assays of predefined ADP-ribosylated peptides containing glutamate (B) or arginine (C) residues. Enzymes tested include TARG1, SARS-CoV2-Mac1, PARP14 MD1, PARP14 MD1 catalytic mutant and ARH1. **(D)** Radioactively labeled polyADPr on automodified PARP1 was incubated with PARG. **(E)** Uncropped SDS-PAGE of purified recombinant deubiquitinases used in this study. Data from this figure can be found in S1 Data.
(PDF)

S3 Fig Purified RNF114/138/166 used in this study. **(A, B)** Uncropped SDS-PAGE of purified recombinant (A) GST-tagged RNF114, RNF125, RNF138 (10-245), and RNF166 and (B) His-tagged RNF114 WT and mutants stained with Coomassie. Red star indicates purified recombinant full-length protein product.
(PDF)

S4 Fig Loss of DTX2 or DTX3L does not alter DNA damage-dependent ADPr. **(A)** western blot analysis of U2OS WT, DTX2KO, DTX3LKO, and HPF1KO cells. Blots were probed with the indicated antibodies. H3 was used as a loading control. **(B–D)** Mean intensity recruitment of (B) YFP-RNF114, (C) YFP-RNF114 ZnF2-3 + UIM, (D) YFP-RNF114 in the presence and absence of olaparib in WT, DTX2KO, or DTX3LKO cells at 240s post-irradiation. **(E)** Western blot analysis of U2OS WT, DTX2KO, and DTX3LKO cells in the absence (white circles) and presence (black circles) of H_2_O_2_. Blots were probed with the indicated antibodies. H3 was used as a loading control. **(F–I)** Mean intensity recruitment of (F) YFP-RNF114, (G) YFP-RNF138, (H) YFP-RNF166, or (I) mCherry-RNF114 complemented with YFP-DTX2 WT or E608R in WT, DTX2KO, or HPF1KO cells at 240s post-irradiation. For all boxplots, limits correspond to the 25th and 75th percentiles, and the bold line indicates the median value. The whiskers extend 1.5 times the interquartile range. *P* values were calculated using an unpaired two-sided Student *t* test, assuming unequal variances. The data for this figure can be found at https://www.ebi.ac.uk/biostudies/studies/S-BIAD2514[74].
(PDF)

S1 Data ADP-ribosyl hydrolases are active on Glu- and Arg-ADPr. Luminescence data collected for AMP-Glo assays in S2 Fig. Data show triplicate raw data and background-subtracted data used in plots.
(XLSX)

S1 Raw Images Uncropped images of Figs 1, 2, 3, and S1–S4. (PDF)

## Abbreviations

ADPr: ADP-ribosylation
ADPr-Ub: ADP-ribosyl-ubiquitin
AMP: adenosine monophosphate
ARHs: ADP-ribosylhydrolases
DUBs: deubiquitinases
FBS: fetal bovine serum
PBST: PBS-Tween 20
PTM: post-translational modification
Ser-ADPr: Serine-ADPr
UIM: ubiquitin-interacting motif
ZnF: zinc finger
