# Supplementary figures and images for: Specificity and recognition of the ADP-ribosyl-ubiquitin modification in the DNA damage response
Source: PLoS Biol. 2026 Apr 2;24(4):e3003747. doi: 10.1371/journal.pbio.3003747 (PMC13061329; doi:10.1371/journal.pbio.3003747)

A

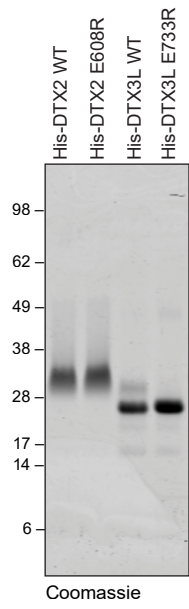

B

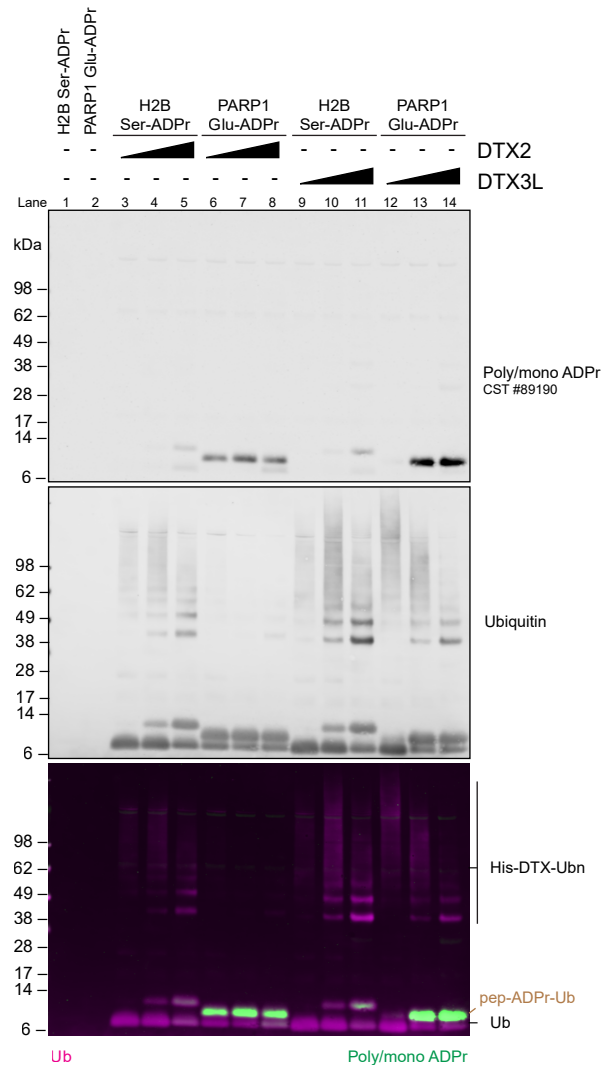

Supplement: S1 Fig — (A) Uncropped SDS-PAGE of purified recombinant His-DTX2 and His-DTX3L WT and mutant RING-DTC domains. (B) PARP1 Glu-ADPr, H2B Ser-ADPr peptides reacted with titrated amounts (0.3, 0.8, 2 µM) of DTX2(RING-DTC) and DTX3L(RING-DTC). Reactions in lanes 3–14 contain E1, UbcH5b, Ub, MgCl2, ATP. ADP-ribosylated peptides were detected with antibodies for ubiquitin or poly/mono-ADPr. ADPr-Ub on peptide substrates are indicated as pep-ADPr-Ub on the merged images. (PDF) [file pbio.3003747.s001.pdf]

A

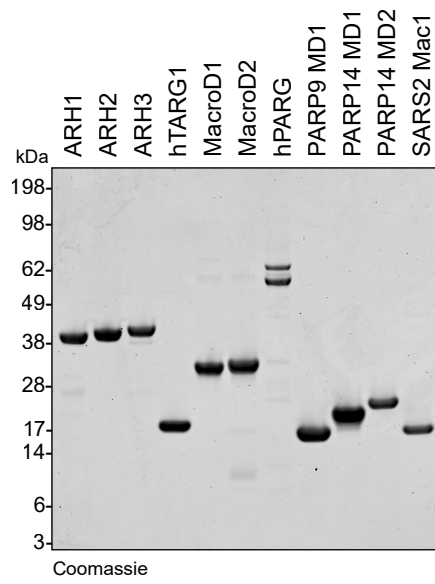

B

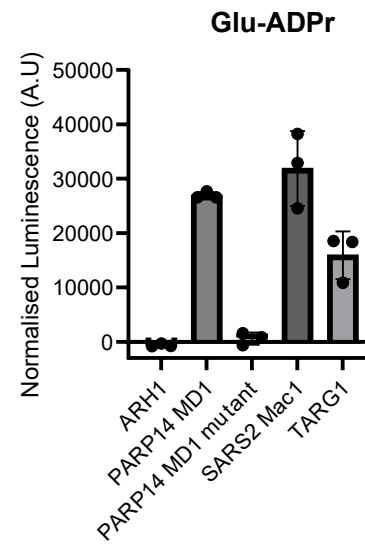

C

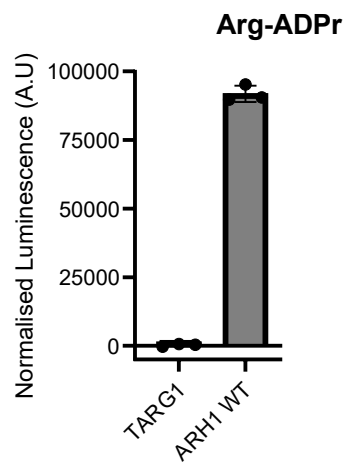

D

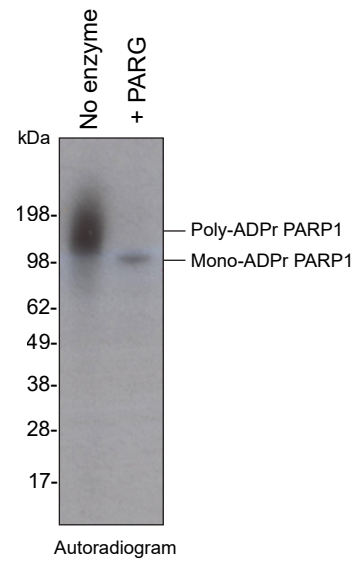

E

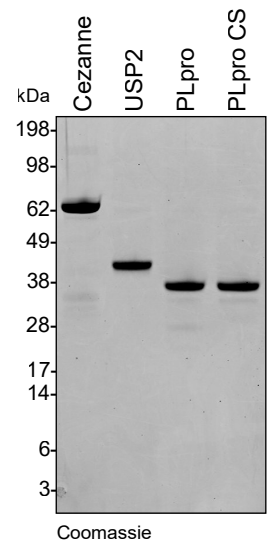

Supplement: S2 Fig — (A) Uncropped SDS-PAGE of purified recombinant ADP-ribosylhydrolases used in this study. (B, C) Hydrolytic assays of predefined ADP-ribosylated peptides containing glutamate (B) or arginine (C) residues. Enzymes tested include TARG1, SARS-CoV2-Mac1, PARP14 MD1, PARP14 MD1 catalytic mutant and ARH1. (D) Radioactively labeled polyADPr on automodified PARP1 was incubated with PARG. (E) Uncropped SDS-PAGE of purified recombinant deubiquitinases used in this study. Data from this figure can be found in S1 Data. (PDF) [file pbio.3003747.s002.pdf]

A

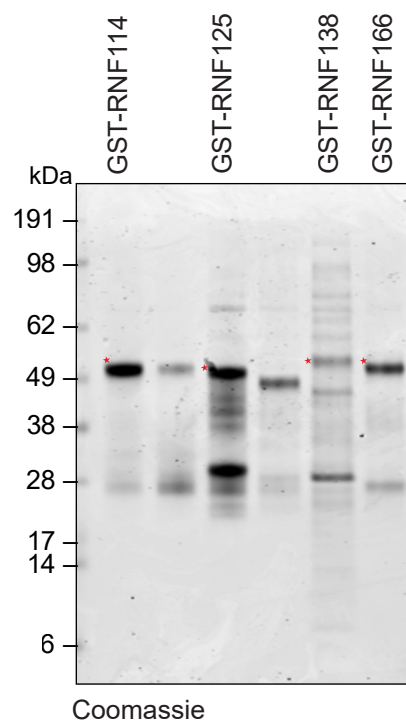

B

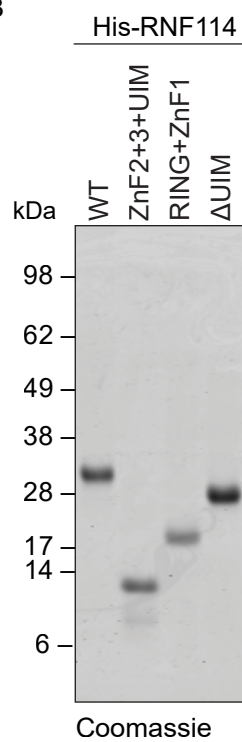

Supplement: S3 Fig — (A, B) Uncropped SDS-PAGE of purified recombinant (A) GST-tagged RNF114, RNF125, RNF138 (10-245), and RNF166 and (B) His-tagged RNF114 WT and mutants stained with Coomassie. Red star indicates purified recombinant full-length protein product. (PDF) [file pbio.3003747.s003.pdf]

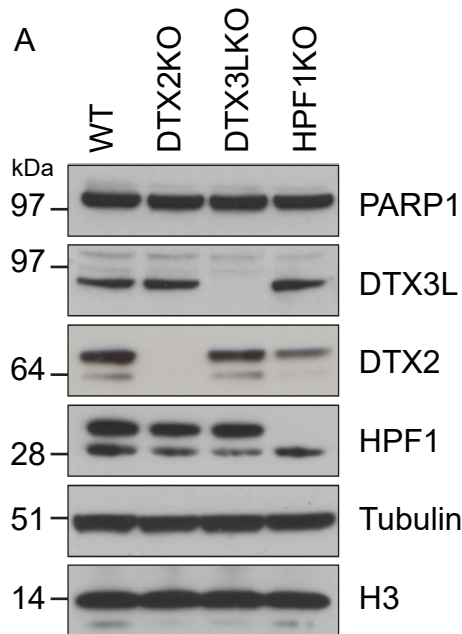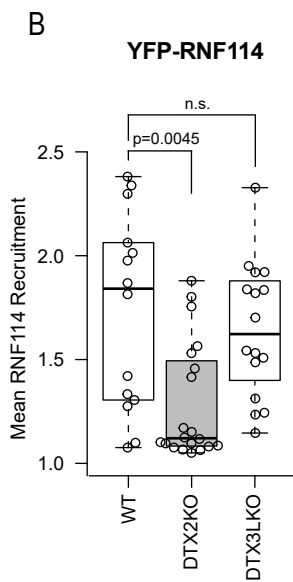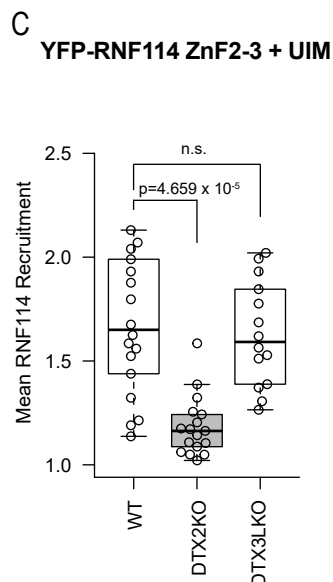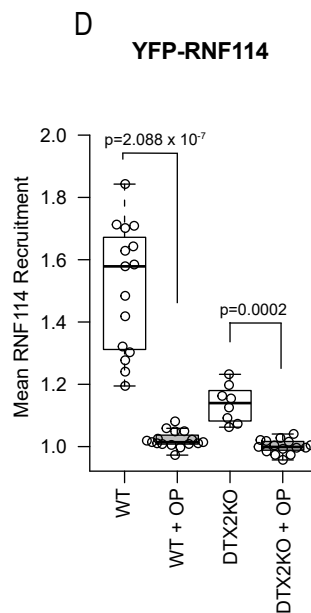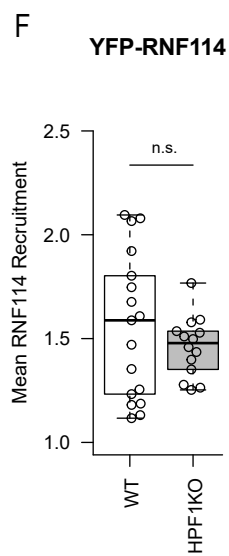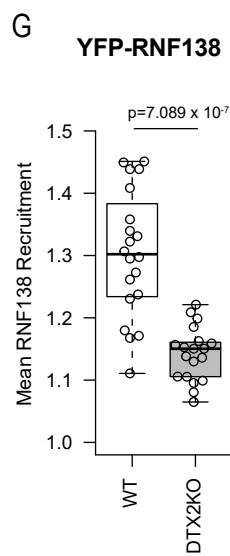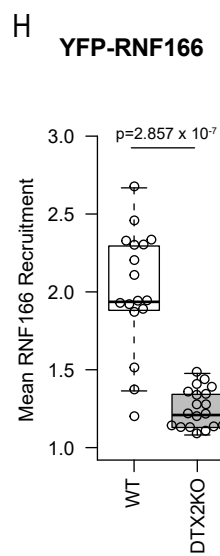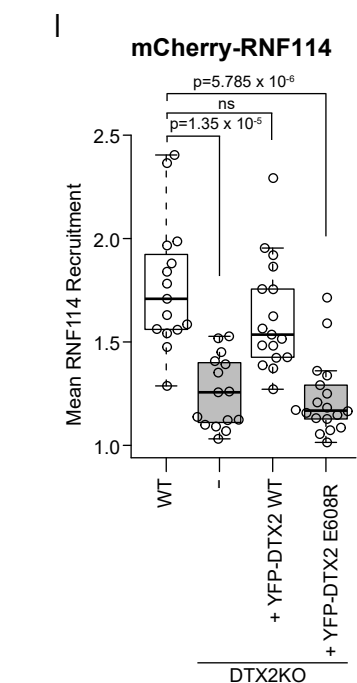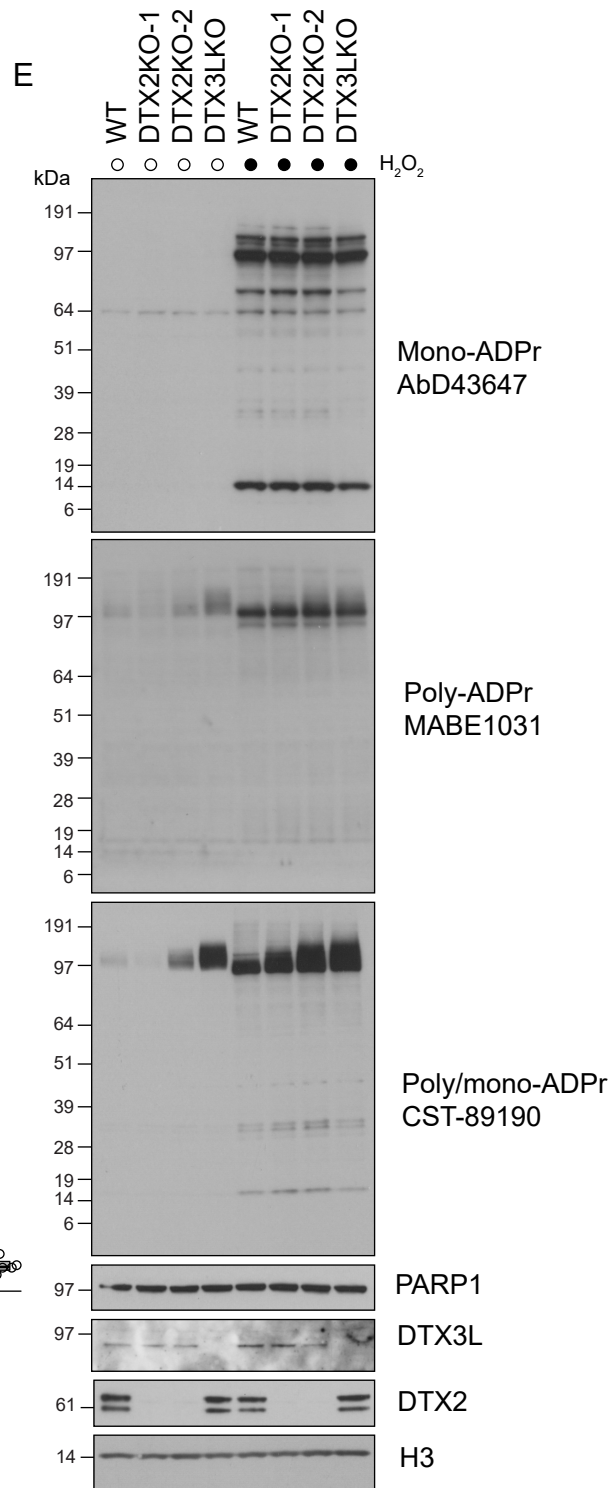

Supplement: S4 Fig — (A) western blot analysis of U2OS WT, DTX2KO, DTX3LKO, and HPF1KO cells. Blots were probed with the indicated antibodies. H3 was used as a loading control. (B–D) Mean intensity recruitment of (B) YFP-RNF114, (C) YFP-RNF114 ZnF2-3 + UIM, (D) YFP-RNF114 in the presence and absence of olaparib in WT, DTX2KO, or DTX3LKO cells at 240s post-irradiation. (E) Western blot analysis of U2OS WT, DTX2KO, and DTX3LKO cells in the absence (white circles) and presence (black circles) of H2O2. Blots were probed with the indicated antibodies. H3 was used as a loading control. (F–I) Mean intensity recruitment of (F) YFP-RNF114, (G) YFP-RNF138, (H) YFP-RNF166, or (I) mCherry-RNF114 complemented with YFP-DTX2 WT or E608R in WT, DTX2KO, or HPF1KO cells at 240s post-irradiation. For all boxplots, limits correspond to the 25th and 75th percentiles, and the bold line indicates the median value. The whiskers extend 1.5 times the interquartile range. P values were calculated using an unpaired two-sided Student t test, assuming unequal variances. The data for this figure can be found at https://www.ebi.ac.uk/biostudies/studies/S-BIAD2514[74]. (PDF) [file pbio.3003747.s004.pdf]
