# Supplementary material for: Specificity and recognition of the ADP-ribosyl-ubiquitin modification in the DNA damage response
Source: PLoS Biol. 2026 Apr 2;24(4):e3003747. doi: 10.1371/journal.pbio.3003747 (PMC13061329; doi:10.1371/journal.pbio.3003747)
Supplement: S1 Raw Images — (PDF) [file pbio.3003747.s006.pdf]

Figure 1A

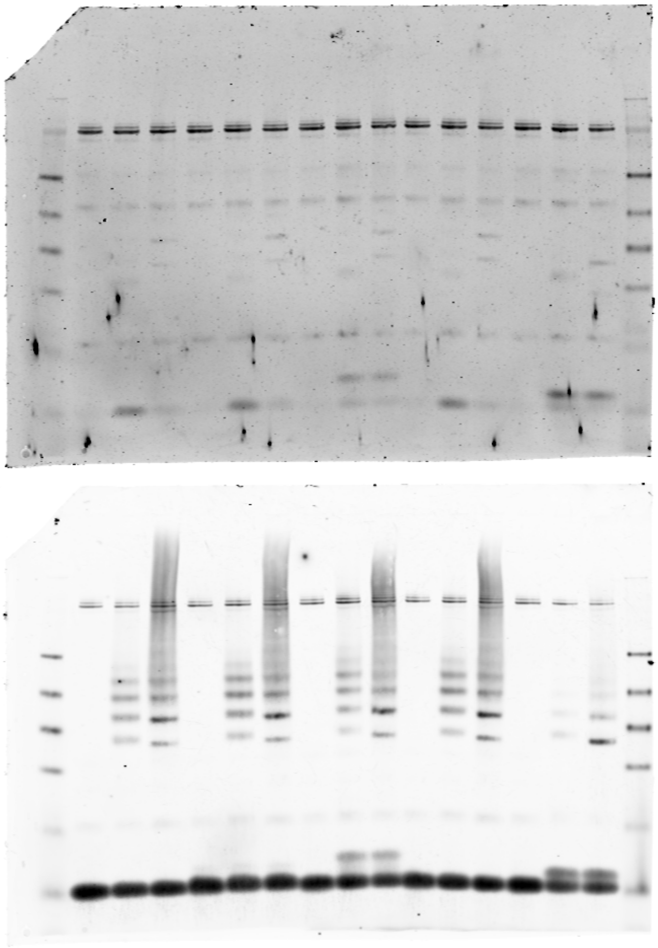

Figure 1D

Figure 1B

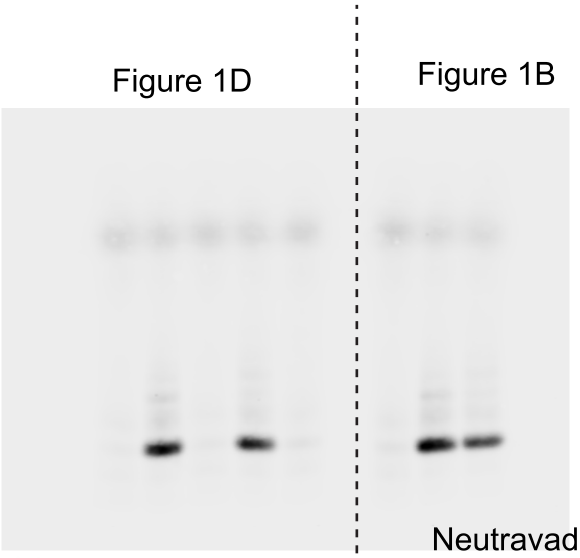

Neutravadin

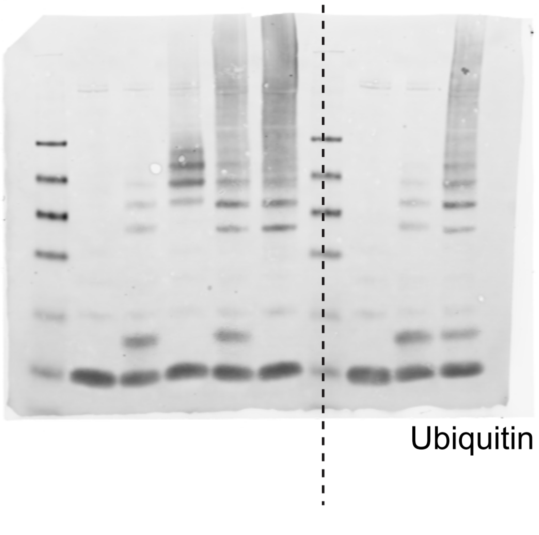

Ubiquitin

Figure 1C

Buffer

PARP1 Glu-ADPr

H2B Ser-ADPr

– DTX2 WT DTX2 E608R DTX3L WT DTX3L E733R – DTX2 WT DTX2 E608R DTX3L WT DTX3L E733R – DTX2 WT DTX2 E608R DTX3L WT DTX3L E733R x

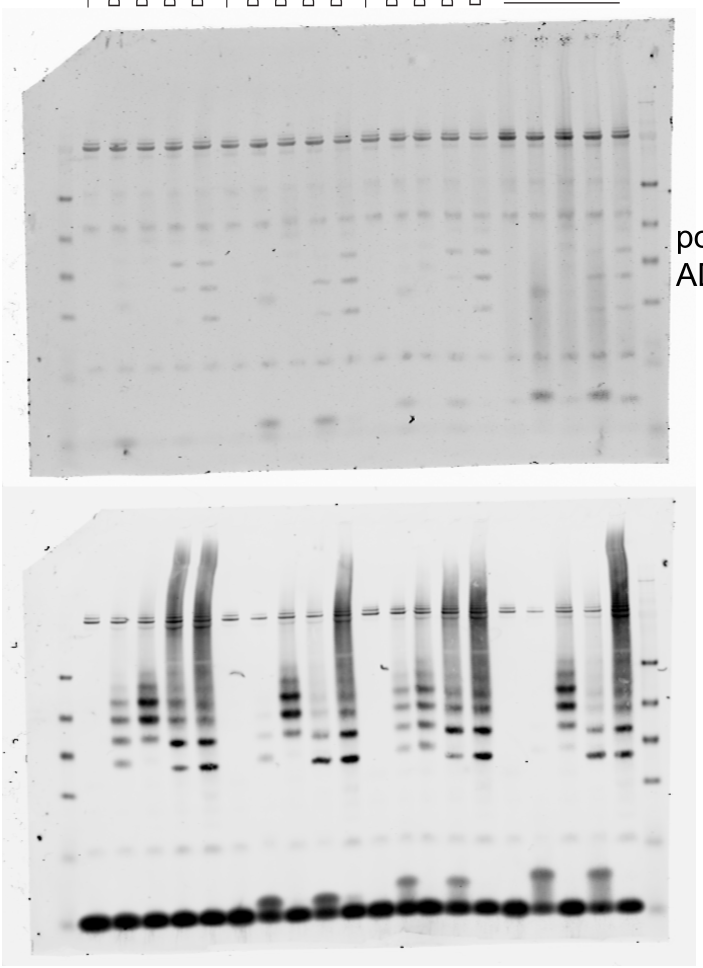

Figure 2A Figure 2C

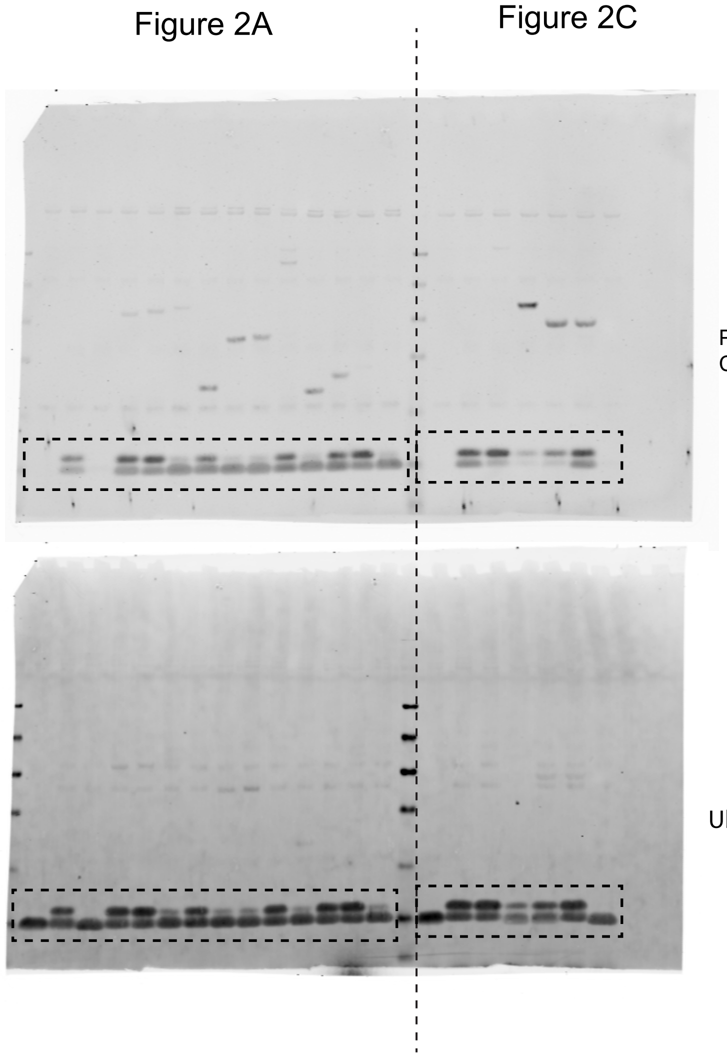

Poly/mono ADPr
CST #89190

Ubiquitin

Figure 2B x

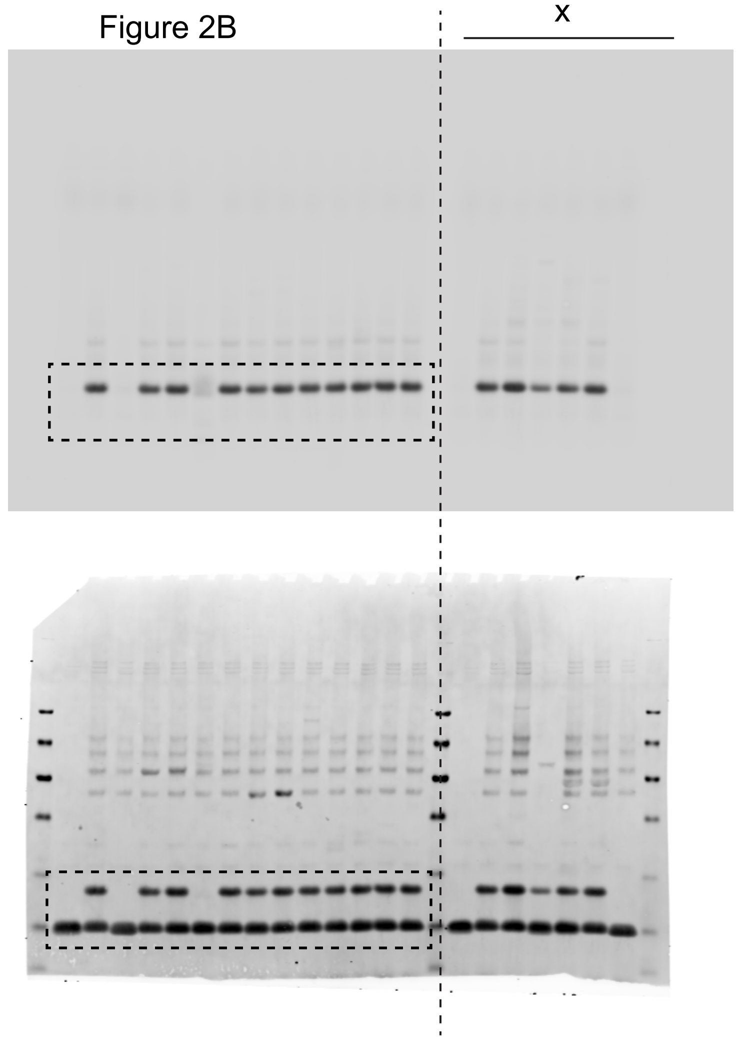

NeutrAvidin

Ubiquitin

Figure 3A

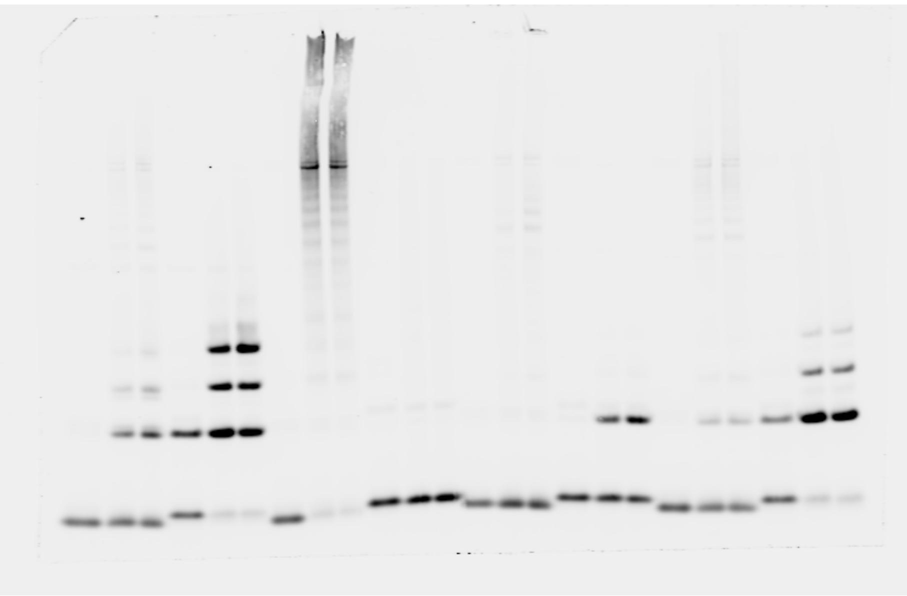

Figure 3C

Figure 3B

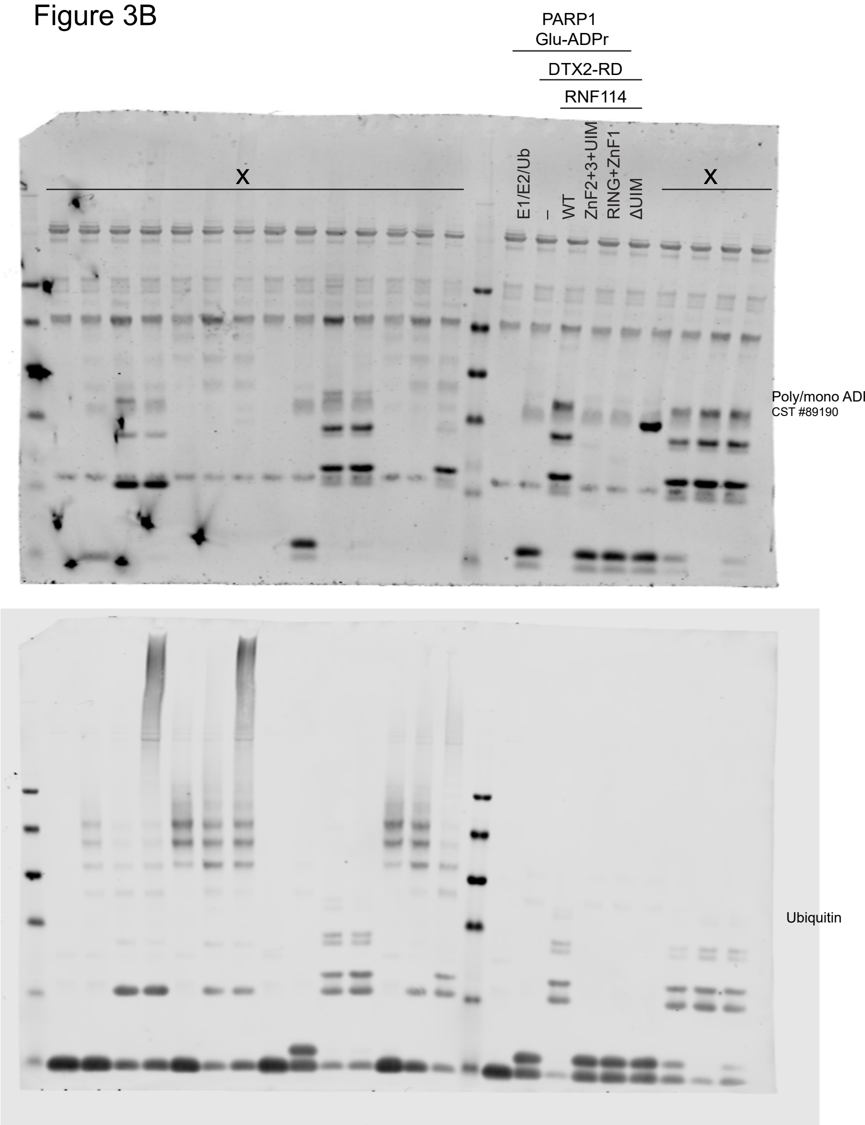

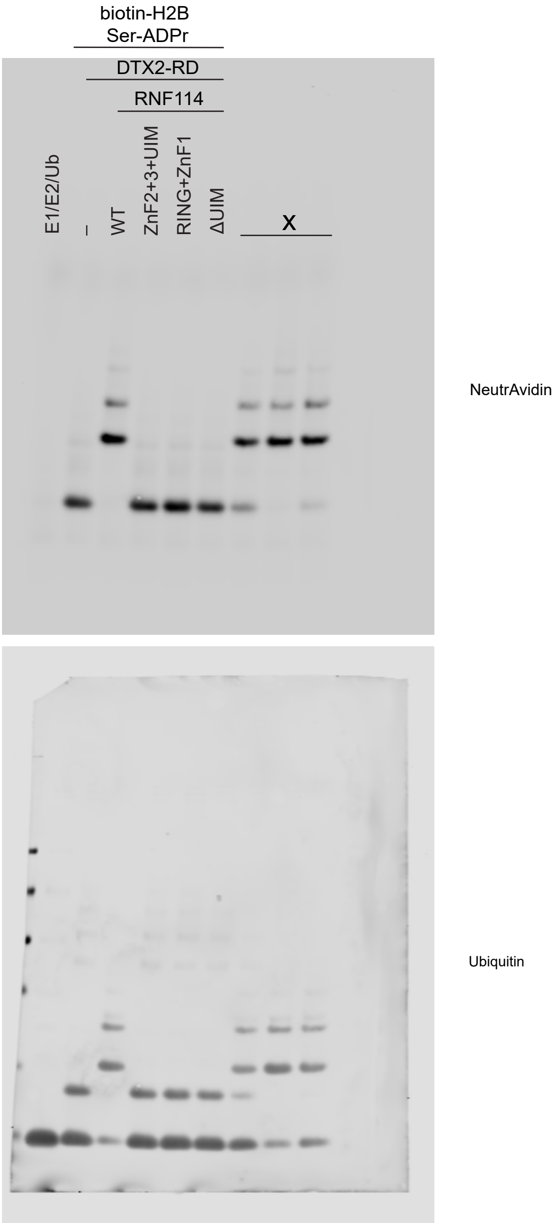

Supp Fig 1A Supp Fig 3B

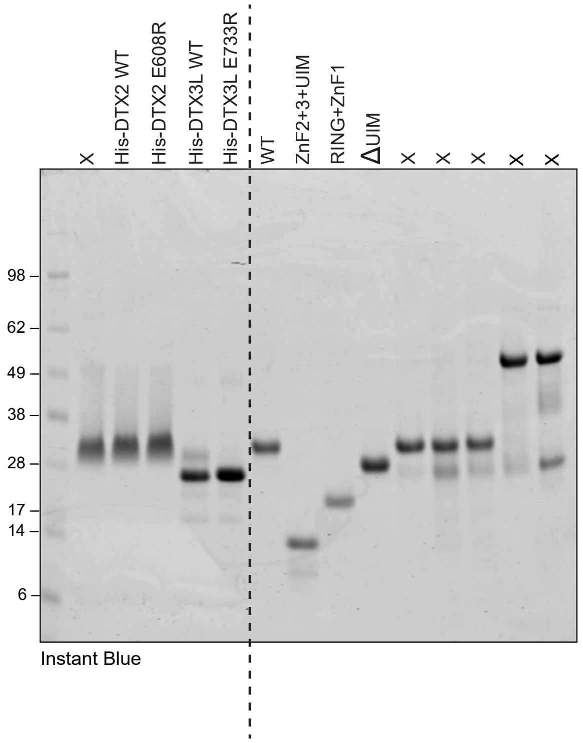

Instant Blue

Supp Fig 3A

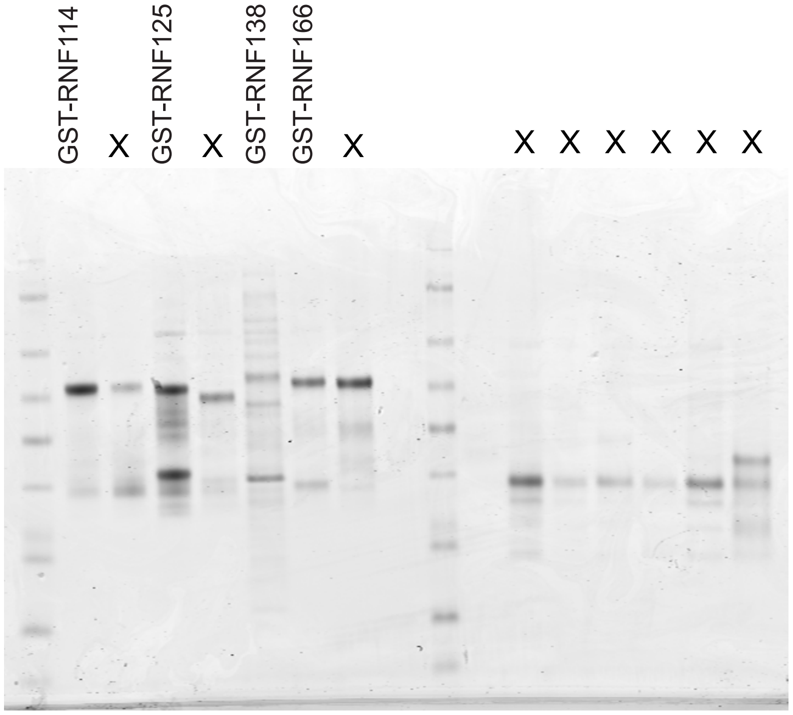

Coomassie

Supp Fig 2A

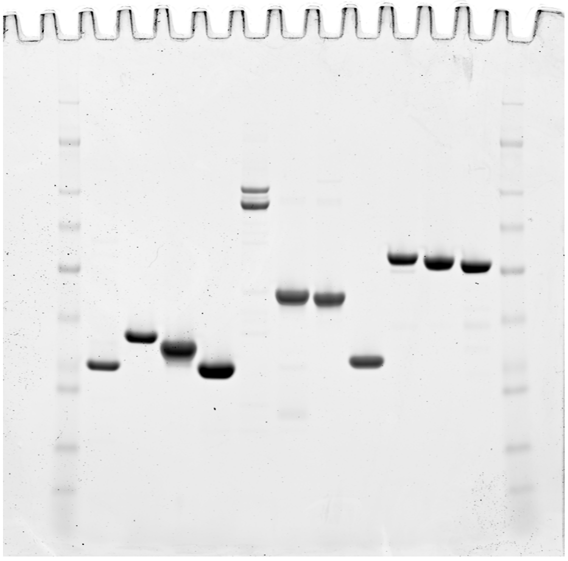
Coomassie

Supp Fig 2D

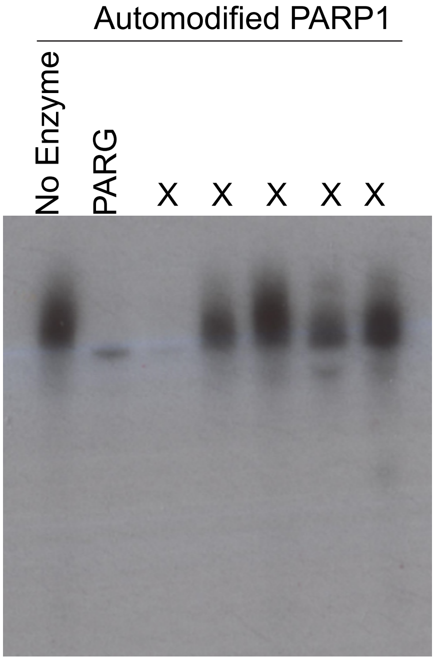

Autoradiograph

Supp Fig 2E

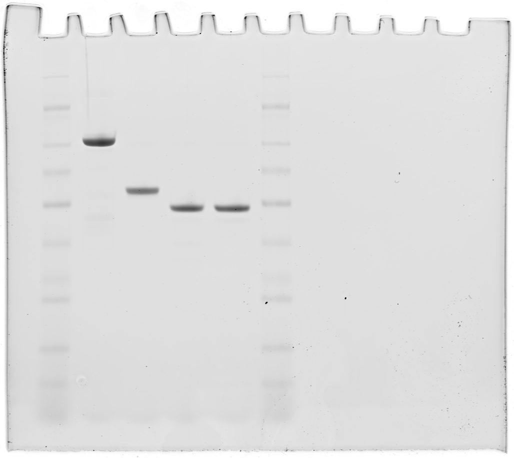
Coomassie

Supp Fig 1 B

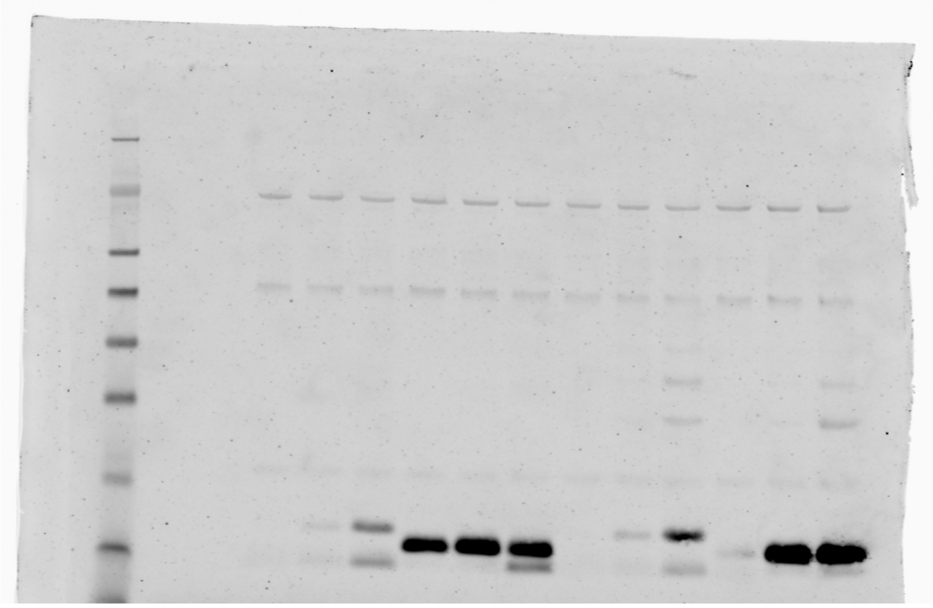

PolyMono ADPr

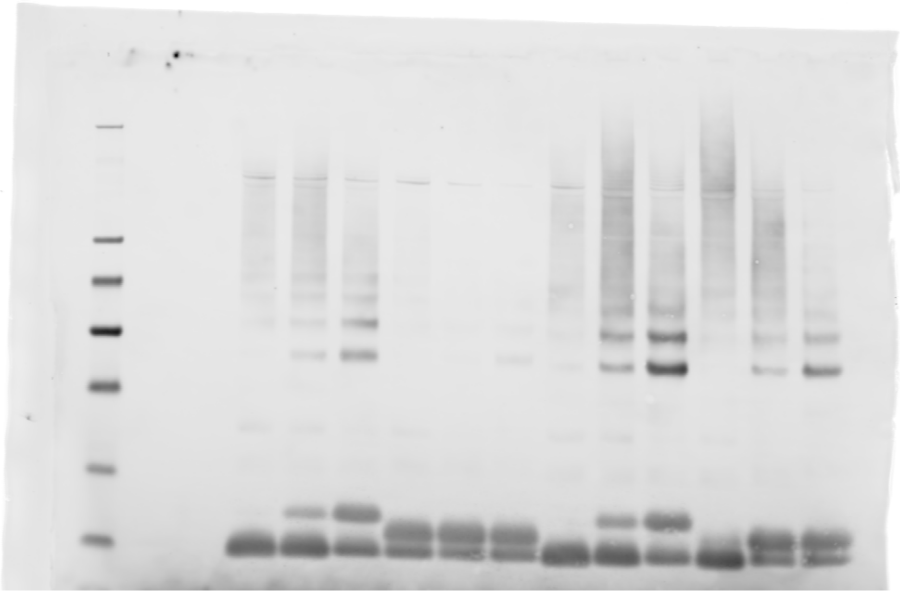

Ubiquitin

Supplemental Fig 4A

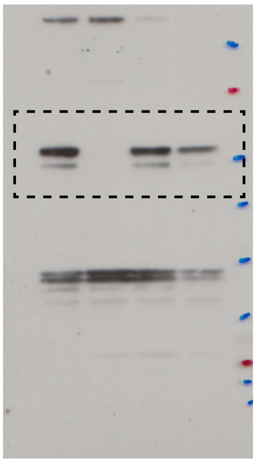

DTX2

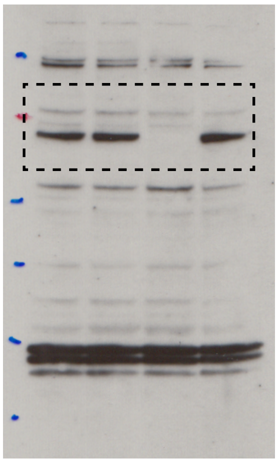

DTX3L

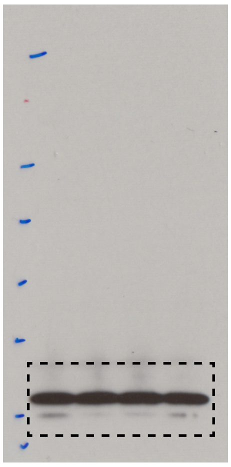

H3

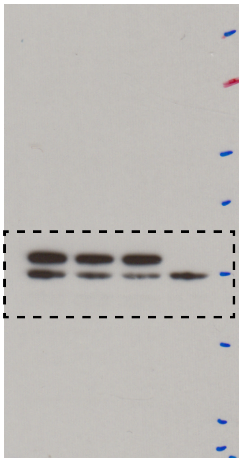

HPF1

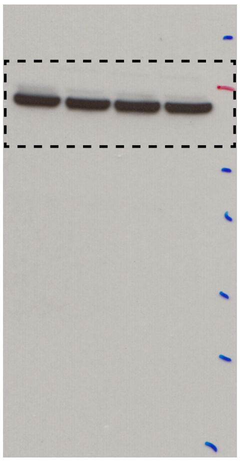

PARP1

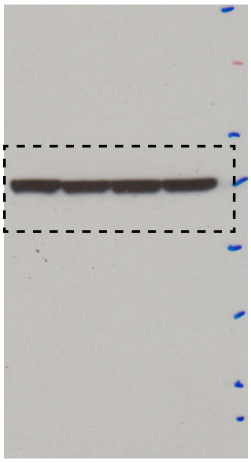

Tubulin

Supplemental Fig 4E

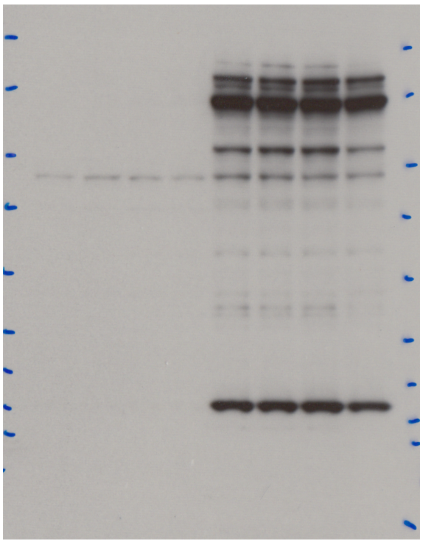

Mono-ADPr

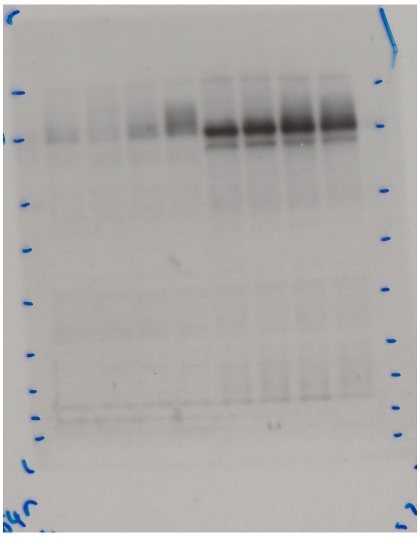

Poly-ADPr

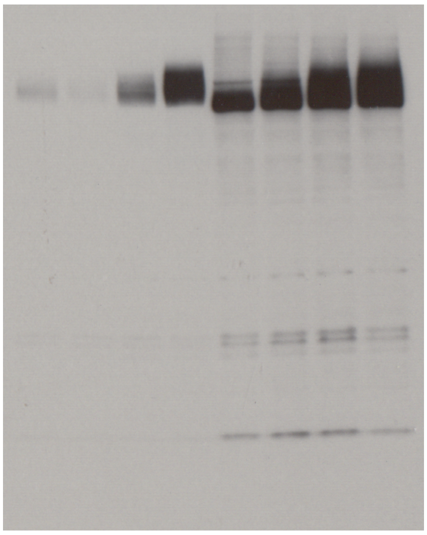

Poly/mono-ADPr

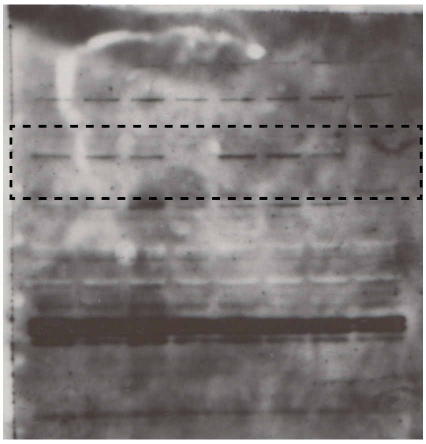

DTX3L

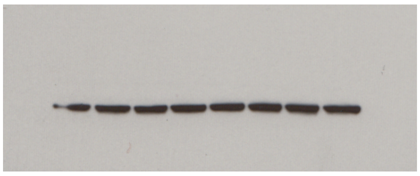

PARP1

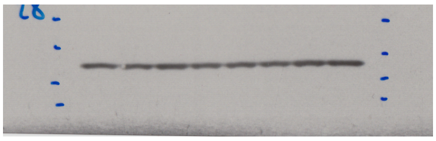

H3

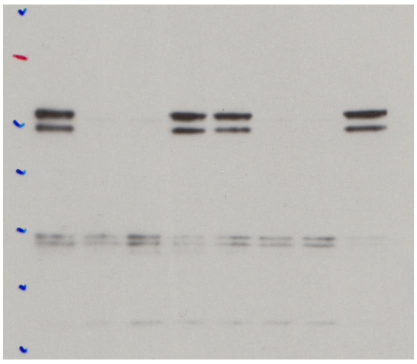

DTX2
